# Sources, toxicity potential, and human health risk assessment of heavy metals-laden soil and dust of urban and suburban areas as affected by industrial and mining activities

**DOI:** 10.1038/s41598-022-12345-8

**Published:** 2022-05-28

**Authors:** Hamed A. Al-Swadi, Adel R. A. Usman, Abdullah S. Al-Farraj, Mohammad I. Al-Wabel, Munir Ahmad, Abdulelah Al-Faraj

**Affiliations:** 1 Soil Science Department, College of Food and Agriculture Sciences, King Saud University, PO Box: 2460, Riyadh, 11451 Saudi Arabia; 2 Department of Soil, Water and Environment, Faculty of Agriculture, Food and Environment, Sana’a University, Sana’a, Yemen; 3 Department of Soils and Water, Faculty of Agriculture, Assiut University, Assiut, 71526 Egypt; 4 Agriculture Engineering Department, College of Food and Agriculture Sciences, King Saud University, Riyadh, Saudi Arabia

**Keywords:** Environmental monitoring, Environmental impact

## Abstract

Sources and levels of heavy metals (HMs) in soil and dust of urban and suburban areas in Riyadh (industrial city) and Mahad AD’Dahab (mining area) cities in Saudi Arabia were reported in this study. Additionally, the concentrations of HMs in different soil particle size fractions (> 250, 63–250 and < 63 µm) were reported. Pollution extent, and ecological and human health risks associated with collected soil and dust samples were explored. Contamination levels of HMs were higher in dust as compared to soil samples at all sites. The average integrated potential ecological risk in dust samples of urban area of Mahad AD’Dahab was 139, and thus characterized as a very-high-risk criterion. Enrichment factor (EF), correlation analyses, and principal component analysis showed that aluminum (Al), cobalt (Co), chromium (Cr), iron (Fe), manganese (Mn), nickel (Ni), titanium (Ti), and zinc (Zn) had mainly the lithogenic occurrence (EF < 2). However, Zn, copper (Cu), and lead (Pb) in Riyadh, and cadmium (Cd), Cu, Zn, and Pb in the Mahad AD’Dahab were affected by industrial and mining activities, respectively, that were of anthropogenic origins (EF > 2). The hazard index values of dust and soil (< 63 µm) samples in both urban and suburban areas in Mahad AD’Dahab were > 1, suggesting non-carcinogenic risk. Therefore, the dust and soil samples from the mined area of Mahad AD’Dahab had a higher pollution levels, as well as ecological and human health risks than those from Riyadh. Hence, the pollution of such residential environments with HMs (especially Cd, Cu, Zn, and Pb) needs to be monitored.

## Introduction

Interest in the characterization of urban soils and dust has increased greatly in the last two decades. Owing to the rapid growth of urban zones accompanied by increase in residential areas, streets, commercial, and industrial zones, increasing pollution levels have been recorded in urban environments^1–3^. Prompt industrialization has resulted in the contamination of terrestrial environments with various pollutants such as heavy metals (HMs). Various sources are responsible for very high concentrations of HMs in different environmental compartments of urban and suburban areas. The elevated levels of HMs in soil could be either of lithogenic or anthropogenic origin^4^. Anthropogenic activities including emissions from vehicles, industrial waste, atmospheric deposition of dust and aerosols, and incinerators have introduced HMs into environments at significant levels^5,6^. Thus, anthropogenic activities are the main reason for HMs contamination of soil as well as airborne dust^7^.

Dust storms are a natural phenomenon in desert ecosystems and their frequency have increased in some parts of the world since 1950s^8^. The dust can be contaminated with HMs from industrial and vehicle emissions^9^. Therefore, it is an important factor for urban pollution, particularly in arid and semi-arid regions of the world such as Saudi Arabia. Saudi Arabia is generally comprised of desert and is known by its hot and dry climate, with the highest average maximum summer temperature. Owing to the vast desert, harsh weather conditions, and low annual precipitation, Saudi Arabia faces many dust storms every year. The dust serves as a medium to accumulate HMs and other atmospheric contaminants emitted through various anthropogenic activities^10^.

There are different routes of consumption of airborne particulate metals by humans, including ingestion, dermal contact, and inhalation^11^. Ingestion of HMs-bearing dust and soil particles can pose a potential risk to human health, especially to children^12^. Therefore, the finest fractions of dust and soil can be used as a good indicators of the bio-accessibility of metals, and are more relevant to human health than whole soil owing to their capacity to adhere to skin or ingested or inhaled^13^. Previously, it has been reported that the bio-accessible fractions of manganese (Mn), nickel (Ni), and zinc (Zn) are higher in urban areas, thereby posing a potentially greater health risk^14^. HMs can have dangerous and toxic effects when they are present above certain concentrations, are not metabolized by the body, and accumulate in the soft tissues^13,15^.

Among the various pathways, soil and dust ingestion is a dangerous route (especially for children), mainly owing to “hand to mouth” activity during outdoor activities with an intake rate of 200 mg soil day^−1^, as indicated by the US-Environmental Protection Agency^16^. For instance, higher blood lead (Pb) values in children have been found to be associated with fine soil and dust particles ingestion^17^. Fine particles tend to adhere more efficiently to hands and thus can easily be ingested into the body^18^. It was found in two European cities i.e., Sevilla and Torino that the availability of HMs in the clay fraction (< 2 μm) was higher than that in other fractions (< 2, 2–10, 10–22, 22–50, and > 50 μm) or whole soils. It indicates that the bio-accessibility of HMs is expected to be higher in the fine fraction of soil. Thus, it is more related to human health than whole soil, which is mainly owing to its ability to adhere to skin or be ingested or inhaled as suspended dust^13^. Therefore, long term exposure to soil and dust contaminated with HMs can cause severe health effects, including lung, kidney, and liver damage as well as cancer. Chromium (Cr) exposure may lead to cancer, Pb exposure could affect cognitive development in children, enzymatic inhibition, as well as nervous and skeletal damage^19^, and cadmium (Cd) exposure could damage kidney, bone, and lungs^20^.

Riyadh has a hot and dry climate with the highest average maximum summer temperature. It in know for vast desert, extreme weather, higher traffic density, and industrial activities, which are causing the accumulation of significant quantities of dust in the city^21^. Al-Rajhi et al.^22^ investigated the levels of HMs in indoor and outdoor dust in Riyadh, and found that the old industrial area had higher levels of HMs. Likewise, El-Desoky et al.^23^ found that outdoor dust is correlated with indoor dust in Riyadh, thereby resulting in greater concentrations of Pb in indoor dust. They further reported that the concentrations of Pb in the blood of 17.8% of children were higher than the global limit (10 µg dL^−1^). Similarly, another city in Saudi Arabia, Mahad AD’Dahab is also facing higher pollution levels of HMs in its environment, which is mainly owing to its closeness to mining activities. Elevated levels of HMs contamination have been observed in sites next to the ground rock landfill in Mahad AD’Dahab^24^. Previously, Al-Farraj et al.^25–27^ have demonstrated that the soil of Mahad AD’Dahab contained very high levels of Cd, copper (Cu), Pb, and Zn. In this context, Al Bakheet et al.^28^ found that the concentrations of Pb, mercury (Hg), and Cd in the blood samples of Mahad AD’Dahab residents were higher than those of Riyadh residents. They also reported that HMs-prone groups are significantly associated with kidney disease, urinary tract disorders, growth disorders, blood diseases, and genetic disorders. Both Riyadh and Mahad AD’Dahab cities have been found to be polluted with HMs through anthropogenic activities such as vehicle transmission, industrial emission, and mining activities; however, the extent of HMs contamination in urban and suburban areas of both cities and their associated ecological and human health risks have not been explored yet.

Therefore, the objectives of this study were to (1) investigate the levels of different HMs in dust as well as different size fractions of soil collected from several urban and suburban areas of Riyadh and Mahad AD’Dahab; and (2) assess the pollution levels, sources, and associated ecological and human health risks posed by HMs in dust and soil (< 63 µm).

## Materials and methods

### Study area, sampling, and analyses

Soil and dust samples were collected from various urban and suburban areas of Riyadh and Mahad AD’Dahab (Supplementary Fig. S1a,b). Nineteen sites in Riyadh and five sites in Mahad AD’Dahab, including urban and suburban areas, were selected for soil and dust sample collection. Soil samples were taken as a compound samples from the same place dust sample was taken. Soil samples were collected at a depth of 0–3 cm. The soil samples were taken, which were affected by the falling dust as a result of industrial and mining activities. Dust samples were collected through a Marble Dust Collector measuring 52.5 × 31.5 cm (Supplementary Fig. S2). The dust samples were collected on a monthly basis from May 2014 to April 2015 and were divided into four groups according to the seasons of collection (summer, autumn, winter, and spring). The dust samples were collected using a soft plastic brush, stored in plastic bags, and transported to the laboratory for analysis. Soil and dust samples were air dried at room temperature (24–25 °C). Soil samples were passed through a 2 mm sieve and then divided into three fractions using sieves ranging as follow (bulk, 63–250 and < 63 µm). The total HMs (Al, Fe, Mn, Zn, Ti, Cu, Cr, Co, Ni, Pb, and Cd) content in the soil and dust was assessed after digestion in a microwave (MARS, CEM Corporation, USA) using a method reported by USEPA 3051^29^ (total-recoverable). Specifically, 0.5 g of each dust or soil sample was place into a Teflon vessel, to which 10 mL of HNO_3_ was added. The vessels were then capped, placed in a microwave, and digested for 10 min according to the USEPA 3051 method. The digested samples were filtered through a 0.45 µm membrane and brought to a total volume of 50 mL with deionized water in a volumetric flask. HMs were measured using inductively coupled plasma optical emission spectrometry (ICP-OES; PerkinElmer Optima 4300 DV, USA). For quality control, soil and dust samples were analyzed in four replicates. Q_test_ was applied to exclude abnormal values at a confidence level of 95%.

### Levels of HMs in soil and dust samples

In this study, contamination levels of HMs in soil and dust were characterized using the pollution index (PI), the integrated pollution index (IPI), the geo-accumulation index (I_geo_), and the potential ecological risk index (RI). The pollution index and the IPI are used to assess environmental quality^30^. The PI is calculated as the ratio of the metal concentration in the sample to the background content of the corresponding metal in the lithosphere (content of the earth’s crust). The following classification of PI was used: PI ≤ 1, low level; 1 < PI ≤ 3, middle level; and PI > 3, high level. The IPI of all measured metals in samples was defined as the mean value of the PI of the metals. The classification of IPI was as follows: IPI ≤ 1, low level; 1 < IPI ≤ 2, middle level; and IPI > 2, high level^31^.

The I_geo_ method was used to calculate the metal pollution levels^32^. The I_geo_ is computed via the following equation.

1 $${I}_{geo}={log}_{2}\frac{Ci}{1.5Bi},$$

where C_i_ is the measured concentration of the metal i and B_i_ is the geochemical background value of the metal. In this study, B_i_ was the background content of the metal i (background in shale)^33^. The 1.5 constant was introduced to minimize the variation of background values. The following classifications were carried out according to I_geo_: unpolluted, I_geo_ ≤ 0; unpolluted to moderately polluted, 0 < I_geo_ ≤ 1; moderately polluted, 1 < I_geo_ ≤ 2; moderately to strongly polluted, 2 < I_geo_ ≤ 3; strongly polluted, 3 < I_geo_ ≤ 4; strongly to extremely polluted, 4 < I_geo_ ≤ 5; and extremely polluted (5 < I_geo_)^32^.

The potential ecological RI originally mentioned by Hakanson^34^ was also calculated to assess the degree of HMs pollution in soil and dust samples using the following equations.

2 $$RI=\sum_{i=1}^{n} Ei,$$

3 $$Ei=Ti fi,\mathrm{ and}$$

4 $$fi= \frac{Ci}{Bi},$$

where RI is the sum of all six risk factors for HMs, E_i_ is the monomial potential ecological risk factor, T_i_ is the metal toxic factor (with the values for each metal in the order of Zn = 1 < Cr = 2 < Cu = Ni = Pb = 5 < Cd = 30)^35^, f_i_ is the metal pollution factor, C_i_ is the concentration of metals in dust, and Bi is a reference value for metals^34^. Different RI classifications of metal pollution are low ecological risk (RI ≤ 150), moderated ecological risk (150 ≤ RI < 300), considerable ecological risk (300 ≤ RI < 600) and high ecological risk (RI ≥ 600).

The EF is a convenient measure for assessing the degree of metal contamination and determining its probable natural and/or anthropogenic sources^36^. For normalization, a reference Fe concentration is used because of its natural abundance. The EF was calculated using the following equation^37^

5 $${EF}_{m}={\left[{C}_{m\left(soil sample\right)}/{C}_{Fe\left(soil sample\right)}\right]}{}\left[{C}_{m\left(earth crust\right)}/{C}_{Fe\left(earth crust\right)}\right],$$

where C_m_ is the content of the examined metal in the soil sample, C_Fe(soil sample)_ is the content of the reference metal (Fe) in the soil sample, C_m(earth crust)_ is the content of the examined metal in the earth’s crust, and C_Fe(earth crust)_ is the content of the reference metal (Fe) in the earth’s crust. In general, EF values much higher than 2 are mainly considered to indicate anthropogenic sources, while values less than 2 predominantly an origin in background soil material. Moreover, the EF also assists in determining the degree of metal contamination. Five contamination categories are recognized based on this factor: EF < 2 indicates deficiency to minimal enrichment; EF = 2–5, moderate enrichment; EF = 5–20, significant enrichment; EF = 20–40, very high enrichment; and EF > 40, extremely high enrichment^38^. In addition, the lithogenic and anthropogenic HMs content was calculated using the following equation^39^.

6 $${\left[M\right]}_{lithogenic}= {\left[Fe\right]}_{sample}\times ({\left[M\right]/\left[Fe\right])}_{lithogenic,}$$

where [M]_lithogenic_ is the metal concentration of lithogenic origin in the sample (mg kg^–1^), [Fe] sample is the total content of Fe in the soil sample (mg kg^–1^), and ($\frac{\left[M\right]}{\left[Fe\right]})lithogenic$ is the ratio of metal concentration to iron concentration in the earth’s crust. Moreover, the anthropogenic HMs content was calculated using the following equation.

7 $${\left[M\right]}_{anthropogenic}= {\left[M\right]}_{total}-{\left[M\right]}_{lithogenic},$$

where [M]_anthropogenic_ is the anthropogenic HMs content and [M]total is the total content of HMs measured in soil samples.

The Distribution factor (DF) has been widely used to assess the distribution of HMs and environmental risks in the different particle size fractions^5,40^. The (DF) index was calculated by Eq. (8)^41^:

8 $$DF= {C}_{fraction}/{C}_{bulk},$$

where C_fraction_ and C_bulk_ (mg kg^−1^) are concentration of HMs in a given fraction and bulk samples, respectively.

### Risk assessment

#### Exposure assessment

Risk assessment is a multi-step procedure of estimating the nature and probability of adverse human health effects that are caused by HMs in an environmental medium^32^. Risk assessment is based on the consideration of human exposure to soil or dust via three different pathways, namely oral intake (ingestion), inhalation, and intake through skin exposure (dermal intake). The average daily doses (ADDs) through ingestion and dermal contact for dust and soil (< 63 µm) were calculated according to the following equations:

9 $${ADD}_{ingestion}=\frac{{C}_{dust}\times {IR}_{ingestion}\times F\times EF\times ED}{BW\times AT},$$

10 $${ADD}_{dermal}=\frac{{C}_{dust}\times SA \times AF\times ABS\times F\times EF\times ED}{BW\times AT}.$$

All the definitions of the parameters and values of the variables for human health risk assessments are presented in Supplementary Table S1.

#### Non-cancer risk assessment

The non-carcinogenic quotients of exposure to HMs in dust and soil (< 63 µm) were calculated. Non-cancer risks are expressed as a hazard quotient (HQ). The HQ is the quotient of the ADD divided by the reference dose of a specific HMs and for the exposure through each pathway. The HQ of each metal was determined by the following equation^42^:

11 $$HQ=\frac{ADD}{RFD}.$$

To assess the overall potential non-cancer risk, the hazard index (HI) was calculated, as follows^5^:

12 $$HI=\sum HQ={HQ}_{ingestion}+{HQ}_{dermal}.$$

The value of HI ≤ 1 indicates that there is no significant risk of non-carcinogenic effects. On the other hand, there is a chance that non-carcinogenic effects may occur when HI > 1, and the probability increases as the value of the HI increases^43^.

#### Cancer risk assessment

The incremental lifetime cancer risk (ILCR) for an individual is estimated by multiplying the slope factor (SF) with the ADD over a lifetime exposure, as determined by Eq. (13).

13 $$ILCR= ADD \times SF.$$

An ILCR value of < 1.0E−06 is considered small, whereas an ILCR of 1.0E−06 to 1.0E−04 is in the range of the acceptable limit and an ILCR of > 1.0E−04 is likely to be harmful to humans^44^.

### Quality assurance and quality control

Great care was taken to avoid any metal contamination during the process of sampling, digestion, and analyses from the beginning of the study. All equipment and containers were soaked in 10% HNO_3_ for 24 h and then rinsed thoroughly in deionized water prior to use. Each soil and dust sample was replicated four times during digestion and analyses. Q_test_ was used for the identification and rejection of outliers and was applied to exclude abnormal readings at a confidence level of 95%. Moreover, three standard reference soils (Till-1, Till-2, and Till-4) were employed for quality control in HMs analyses in soil (Supplementary Table S2). Standard solutions with known concentrations were simultaneously analyzed in the experiments after each set of 10 samples, to ensure the analytical performance of the ICP-OES apparatus. The imprecision of the method was computed as the relative standard deviation targeted at ≤ 5%. The detection limits of ICP-OES were < 0.1 µg L^–1^ for Cd and Fe and 1 µg L^–1^ for Al, Ti, Co, Cu, Cr, Pb, Mn, Ni, and Zn. Values of the studied metals that were below the detection limits of ICP-OES were rejected. The recovery % of HMs was calculated according to the following equation.

14 $$Recovery\%=({C}_{ex}/{C}_{ref})\times 100,$$

where C_ex_ is the HMs concentration extracted by solution (mg kg^–1^) and C_ref_ is the concentration of HMs in the reference soil “Till” (mg kg^–1^).

Supplementary Table S2. Recovery of HMs content in the three certified reference materials (Till 1, Till 2, and Till 4) digested using the EPA 3051 method.

## Results and discussion

### Distribution of heavy metal concentrations in the soil particle size fraction

Supplementary Table S3a,b show the minimum, maximum, and average concentrations of HMs in the different particle size fractions of urban and suburban soils. The HMs in the different fractions of urban and suburban soils were regularly distributed. Among the particle size fractions, the total content of most of the investigated HMs tended to increase as the size of the soil particles decreased. The highest content was generally pronounced for the particle size fraction of < 0.63 μm. The highest amount of metals that accumulated in the fine particles of < 63 μm could be explained by their high reactivity and their affinity toward HMs^45^. According to some researchers, HMs are often accumulated in the fine fraction, such as clay particles that act as metal sorbents, which is mainly due to their high surface area and negative surface charge^40,45^. It was generally observed that the total HMs concentrations in the mined soils of Mahad AD’Dahab were higher than those in the industrial activity-impacted soils of Riyadh. In this context, urban soils are often contaminated with HMs owing to anthropogenic sources. Previously, it has been reported that mining activities can result in significant metal accumulation in environmental compartments^46^. Alike, several studies found that mining operations are significant sources of HMs contamination in soils and higher contents of HMs in mining-impacted soils result from long-term activities^47,48^.

The Distribution factor (DF) has been widely used to assess the distribution of HMs and environmental risks in the different particle size fractions^5,40^. Supplementary Fig. S3a,b show the minimum, maximum, and average DFs for HMs in the different particle size fractions of urban and suburban soils. These obtained DF values indicated a greater metal accumulation in the finer fraction (< 63 µm) compared with that of larger size fraction (63–250 μm). Our data were in line with previous findings on the preferential partitioning of HMs to fine soil particle size fractions^5,40,49^. This can be explained by the larger surface area of the fine particles, which enhances the adsorption capacity of the fine fraction. Additionally, finer soil particles can have higher contents of secondary clay minerals, which are very strong sorbents for HMs^49,50^. On the contrary, the coarser fractions of sand and silt can have a higher content of the primary mineral quartz (e.g., SiO_2_), thereby leading to lower sorption capacity. It could be concluded that the fine particle fractions, especially the clay fraction, accumulated higher concentrations of HMs than the coarse fractions, thereby causing potential harm to human health and the environment^50^.

Supplementary Table S4 shows the Pearson correlation between the HM concentration in dust and soil < 63 µm over all the investigated areas. The Pearson correlation showed that there was a significant correlation between the total concentration of most HMs in dust and soil < 63 µm. The Pearson correlation was 0.55 for Cd, 0.67 for Cu, 0.53 for Fe, 0.66 for Mn, 0.52 for Pb, 0.55 for Ti, and 0.44 for Zn.

### Heavy metals content in soil and dust samples

Table 1 shows the minimum, maximum, and mean concentrations of total HMs in the bulk soil samples collected from the investigated sites in the urban and suburban areas of Riyadh and Mahad AD’Dahab. The results indicated that the HMs content varied according to the metal type, sampling site, and study area. Generally, it was observed that the total HM concentrations in urban areas were higher than those detected in suburban areas. Among the two localities, the soil samples collected from Mahad AD’Dahab sites had the highest metal concentrations. For instance, the average HMs concentration in soil samples collected from urban Riyadh and Mahad AD’Dahab amounted to 5410 and 14,100 for Al, 0.012 and 0.197 for Cd, “nd” and 0.273 for Co, 9.08 and 13.2 for Cr, 0.702 and 18.5 for Cu, 4710 and 12,900 for Fe, 81.5 and 308 for Mn, 4.41 and 8.37 for Ni, 5.68 and 13.9 for Pb, 117 and 588 for Ti, and 13.7 and 54.3 for Zn (all in mg kg^–1^), respectively.

**Table 1:** Minimum, maximum and average content of heavy metals in soil samples of study area and average content of heavy metals values for world and Netherland soil.

Soil	Heavy metals (mg kg^−1^)										
	Al	Cd	Co	Cr	Cu	Fe	Mn	Ni	Pb	Ti	Zn
**Urban Riyadh**											
Max	12,700	0.099	0.00	31.4	4.41	9990	166	23.80	12.60	240	31.3
Min	2450	0.000	0.00	1.49	0.00	2760	50	0.00	1.28	24	4.7
Average	5410	0.012	0.00	9.08	0.70	4710	82	4.41	5.68	117	13.7
**Suburban Riyadh**											
Max	7650	0.000	2.22	5.94	2.59	9650	163	9.63	4.96	200	19.2
Min	3060	0.000	0.00	4.45	0.00	3600	58.6	3.04	4.38	113	3.36
Average	5360	0.000	1.11	5.19	1.30	6630	111	6.34	4.67	156	11.3
**Urban Mahad AD’Dahab**											
Max	17,800	0.395	0.55	18.4	32.80	15,100	392	14.30	22.20	613	90.0
Min	10,100	0.000	0.00	7.98	4.12	10,600	223	2.38	5.57	563	18.5
Average	14,100	0.197	0.27	13.2	18.50	12,900	308	8.37	13.90	588	54.3
**Suburban Mahad AD’Dahab**											
Max	12,800	0.000	0.00	12.6	17.30	14,600	378	10.90	6.28	987	38.5
Min	8480	0.000	0.00	5.94	6.97	10,300	234	3.80	4.39	631	17.1
Average	10,600	0.000	0.00	9.27	12.10	12,500	306	7.35	5.34	809	27.8
**Common range**^**a**^											
Max	300,000	0.700	40	1000	100	550,000	3000	500	200	10,000	300
Min	10,000	0.010	1	1	2	7000	20	5	2	1000	10
Average	71,000	0.060	8	100	30	38,000	600	40	10	4000	50
**Background in shale**^**b**^											
Average	80,000	0.300	19	90	45	47,200	850	68	20	4600	95
World (av^c^)											
Average		1.10	6.9	42	14		418	18	25	–	62
**Dutch**^**d**^											
OV		0.80	9	100	36		–	35	85		140
AV		12	240	380	190			210	530		720

^a^Lindsay^50^. ^b^Turekian and Wedepohl^33^. ^c^Average concentrations for world soils (Huang et al.^75^). ^d^VROM  ^76^.

In the Riyadh area, the highest total concentrations of most HMs were found at site 16 (with the exception of Cu, Cd, and Co). In the Mahad AD’Dahab area, the highest total concentrations of Cd, Cu, Pb, and Zn were detected at site 3, which is close to the mining area. Moreover, the highest concentrations of Al, Co, Cr, Fe, Ni, and Ti were recorded at site 2.

In Saudi Arabia, quality guidelines for soil HMs have not been established. Therefore, in the current study, the concentrations of HMs in soil samples were compared with other guidelines, including the common range in the earth’s crust, the average concentrations in world soils, the average shale values, and the Dutch optimum and Act target values, as shown in Table 1. The concentrations of most investigated HMs were lower than their corresponding values of the common range in soil according to Ref.^51^. However, in urban Mahad AD’Dahab sites, the average concentrations of Cd, Pb, and Zn were higher than their corresponding values of the common range (0.06, 10, and 50 mg kg^–1^, respectively). Moreover, the maximum and average concentrations of Cu in urban Mahad AD’Dahab sites were higher than the average concentration in world soils.

Supplementary Table S5 shows the average metal concentrations in dust samples in relation to season. Generally, the highest average values of most of the HMs were detected in the spring season in urban and suburban areas of Riyadh and suburban areas of Mahad AD’Dahab. However, in urban areas of Mahad AD’Dahab, the highest average values of most HMs were found in the winter season. The variation in the order of the highest metal levels between these two seasons (winter and spring) could be explained by changes in meteorological conditions.

Table 2 shows the comparison of the obtained average metal concentrations with those reported for other countries. The comparison of HMs levels in the collected dust samples with those of other countries showed that the average content of HMs in the investigated sites in the current study were lower than those of most other cities (e.g., Riyadh (Saudi Arabia), Khamees-Mushait (Saudi Arabia), Jeddah (Saudi Arabia), Middle and South of Iraq, Kermanshah (Iran), Rafsanjan SE (Iran), Shangqing (China), Xi’an (China), Hong Kong (China), Oslo and Madrid). However, they were higher than those measured in Luanda (Angola) (especially for Al, Co, Cr, Ni, Ti, and Zn).

**Table 2:** Average concentrations of heavy metals (mg kg^−1^) in dust of urban and suburban areas and their comparison with those reported for other countries.

Dust	Al	Cd	Co	Cr	Cu	Fe	Mn	Ni	Pb	Ti	Zn	Digestion	References
Riyadh (Urban)	10,400	0.10	4.50	29.7	24.0	12,900	210	26.7	16.1	350	599	HNO_3_	In this study
Riyadh (Suburban)	9440	0.00	2.56	24.3	10.0	11,500	179	21.9	8.6	314	603	HNO_3_	In this study
Mahad AD’Dahab (Urban)	13,900	0.69	8.4	30.0	127	18,500	357	29.3	38.8	803	982	HNO_3_	In this study
Mahad AD’Dahab (Suburban)	10,200	0.12	4.7	18.6	24.3	15,000	294	20.0	7.4	592	666	HNO_3_	In this study
Riyadh (Saudi Arabia)	12,057	–	17.7	46.6	117	13,160	199	20.3	96.3	–	101	HNO_3_:HF:HCl:H_2_O_2_	^65^
Khamees-Mushait (Saudi Arabia)	–	1.16	34.2	186.5	49.4	62,735	803	–	126.4	–	118	HCl:HNO_3_:HF	^66^
Jeddah (Saudi Arabia)	–	7.46	11.7	65.4	139.1	12,449	551	51.3	141.0	–	488	HNO_3_ + HCl HCl/HNO_3_	^67^ ^68^
Middle and South (Iraq)	–	1.33	–	–	–	33,800	–	106.4	76.9	–	216		
Kermanshah (Iran)	–	–	–	73.7	47.6	–	–	119.5	–	–	210.3	HCl/HNO_3_	^69^
Rafsanjan, SE Iran		3.1		18.4	791.4			28.4	123.1		252.6	HF:HNO_3_	^70^
Changqing (China)	–	–	16.4	1591.8	178.2	–	346.5	40.2	1586	–	1918.8		^2^
Xi’an (China)	–	–	19.4	–	102.7	–	581	56.7	266.3	3787	798		^71^
Hong Kong (China)	20,400	–	9.5	124.0	110.0	14,100	594	28.6	120	2370	3840		^72^
Olso	59,527	–	19.0	–	123.0	51,452	833	41.0	180	7452	476	HNO_3_ + HClO_4_ + HF	^73^
Madrid	43,800	–	3.0	61.0	188.0	19,300	362	44.0	1927	1100	476	HNO_3_ + HClO_4_ + HF	^73^
Luanda (Angola)	4839	1.1	2.9	26.0	42.0	11,572	258	10.0	351	107	317	HNO_3_ + HCl	^74^

These results indicated that soil and dust samples were contaminated with Cd, Pb, Cu, and Zn. The highest levels of these HMs in top 3 cm soil layer could be due to the accumulation of HMs-laden dust particles. Jian et al.^52^ has previously reported the contamination of topsoil potentially hazardous elements such HMs. It was suggested that industrial and agricultural activities, traffic emissions and natural sources were responsible for topsoil contamination^52^. Likewise, Wang et al.^53^ collected samples from roadside dust and found that > 90% of dust contained higher levels of HMs such as Cd, Cu, Hg, Pb, As, and Zn, thus posing serious health risks to ecosystem and human health. Similar results were reported by Cai et al.^54^ for soil samples, which reported the contamination of soil by Cd, Pb and Zn through agricultural practices and traffic activities.

### Pollution and ecological indices and sources of HMs in soil and dust samples

The calculated data pertaining to the PI, Igeo, EF, Ei, and RI parameters for all HMs in soil and dust samples from urban and suburban areas of Riyadh and Mahad AD’Dahab are presented in Tables 3, 4, 5 and 6. It was generally observed that the values of pollution and ecological indices of all the analyzed HMs were higher in dust samples vs. soil samples. The calculated data of PI for all HMs of soil and dust samples from urban and suburban areas of Riyadh and Mahad AD’Dahab are presented in Table 3. The results showed that all soil samples collected at Riyadh sites had maximum, minimum, and mean PI values < 1, indicating a low level of pollution with HMs^54^. However, the soil samples collected at urban sites of Mahad AD’Dahab had maximum PI values of 2.0 for Cd, 1.4 for Pb, and 1.1 for Zn, indicating a moderate level of pollution with these HMs. For dust samples, compared with soil samples, the average PI values for Zn were higher than 3, suggesting a high pollution level (Table 3). The urban dust samples collected at Mahad AD’Dahab showed moderate pollution levels regarding Cu (mean PI value = 1.81) and Pb (mean PI value = 2.42), and high pollution levels regarding Cd (mean PI value = 3.45) and Zn (mean PI value = 12.0). Similarly, dust samples collected at Riyadh had average PI values of 7.48 and 7.54 for Zn in urban and suburban areas, respectively; therefore, they exhibited a high pollution level.

**Table 3:** The calculated data of the PI and IPI for heavy metals of soil and dust samples.

Soil	Al	Cd	Co	Cr	Cu	Fe	Mn	Ni	Pb	Ti	Zn	
	PI											IPI
**Urban Riyadh**												
Max	0.2	0.5	0.0	0.2	0.1	0.2	0.2	0.2	0.8	0.0	0.4	0.2
Min	0.0	0.0	0.0	0.0	0.0	0.1	0.1	0.0	0.1	0.0	0.1	0.0
Average	0.1	0.1	0.0	0.0	0.0	0.1	0.1	0.0	0.4	0.0	0.2	0.1
**Suburban Riyadh**												
Max	0.1	0.0	0.1	0.0	0.0	0.2	0.2	0.1	0.3	0.0	0.2	0.1
Min	0.0	0.0	0.0	0.0	0.0	0.1	0.1	0.0	0.3	0.0	0.0	0.1
Average	0.1	0.0	0.0	0.0	0.0	0.1	0.1	0.1	0.3	0.0	0.1	0.1
**Urban Mahad AD’Dahab**												
Max	0.2	2.0	0.0	0.1	0.5	0.3	0.4	0.1	1.4	0.1	1.1	0.6
Min	0.1	0.0	0.0	0.0	0.1	0.2	0.2	0.0	0.3	0.1	0.2	0.1
Average	0.2	1.0	0.0	0.1	0.3	0.3	0.3	0.1	0.9	0.1	0.7	0.3
**Suburban Mahad AD’Dahab**												
Max	0.2	0.0	0.0	0.1	0.2	0.3	0.4	0.1	0.4	0.2	0.5	0.2
Min	0.1	0.0	0.0	0.0	0.1	0.2	0.3	0.0	0.3	0.1	0.2	0.1
Average	0.1	0.0	0.0	0.0	0.2	0.2	0.3	0.1	0.3	0.1	0.3	0.2
**Dust**												
**Urban Riyadh**												
Max	0.25	5.19	0.24	0.29	0.87	0.44	0.41	0.52	2.63	0.10	37.8	3.88
Min	0.09	0.00	0.01	0.10	0.16	0.18	0.16	0.16	0.31	0.02	1.26	0.29
Average	0.13	0.51	0.11	0.15	0.34	0.25	0.23	0.27	1.01	0.06	7.48	0.96
**Suburban Riyadh**												
Max	0.18	0.00	0.09	0.18	0.23	0.31	0.28	0.28	1.06	0.08	14.4	1.51
Min	0.06	0.00	0.00	0.06	0.10	0.15	0.14	0.11	0.35	0.02	1.25	0.21
Average	0.12	0.00	0.06	0.12	0.14	0.23	0.20	0.22	0.54	0.05	7.54	0.84
**Urban Mahad AD’Dahab**												
Max	0.21	9.15	0.25	0.19	2.96	0.43	0.45	0.37	4.91	0.18	19.60	2.90
Min	0.14	0.49	0.18	0.12	0.78	0.32	0.35	0.24	0.99	0.10	5.00	0.80
Average	0.17	3.45	0.21	0.15	1.81	0.36	0.40	0.29	2.42	0.13	12.3	1.90
**Suburban Mahad AD’Dahab**												
Max	0.24	0.83	0.25	0.18	0.81	0.51	0.62	0.38	0.99	0.16	16.70	1.90
Min	0.08	0.49	0.05	0.04	0.15	0.18	0.19	0.09	0.17	0.06	2.77	0.40
Average	0.13	0.62	0.12	0.09	0.35	0.29	0.33	0.20	0.46	0.10	8.31	1.00

**Table 4:** The calculated data of the I_geo_ for heavy metals of soil and dust samples.

Soil	*I*_*geo*_											Average
	Al	Cd	Co	Cr	Cu	Fe	Mn	Ni	Pb	Ti	Zn	
**Urban Riyadh**												
Max	− 3	0	0	− 2	0	− 3	− 3	0	− 1	− 5	− 2	− 2
Min	− 6	0	0	− 7	0	− 5	− 5	0	− 5	− 8	− 5	− 5
Average	− 5	0	0	− 4	0	− 4	− 4	0	− 3	− 6	− 4	− 3
**Suburban Riyadh**												
Max	− 4	0	0	− 5	0	− 3	− 3	− 3	− 3	− 5	− 3	− 3
Min	− 5	0	0	− 5	0	− 4	− 4	− 5	− 3	− 6	− 5	− 4
Average	− 5	0	0	− 5	0	− 4	− 4	− 4	− 3	− 6	− 4	− 3
**Urban Mahad AD’Dahab**												
Max	− 3	0	0	− 3	− 1	− 2	− 2	− 3	0	− 3	− 1	− 2
Min	− 4	0	0	− 4	− 4	− 3	− 3	− 5	− 2	− 4	− 3	− 3
Average	− 3	0	0	− 3	− 3	− 2	− 2	-4	− 1	− − 4	− 2	− 3
**Suburban Mahad AD’Dahab**												
Max	− 3	0	0	− 3	− 2	− 2	− 2	− 3	− 2	− 3	− 2	− 2
Min	− 4	0	0	− 5	− 3	− 3	− 2	− 5	− 3	− 3	− 3	− 3
Average	− 4	0	0	− 4	− 3	− 3	− 2	− 4	− 3	− 3	− 2	− 2
**Dust**												
**Urban Riyadh**												
Max	− 3	1	− 2	− 1	− 1	− 2	− 2	− 1	0	− 4	3.53	− 1
Min	− 4	− 1	− 6	− 3	− 3	− 3	− 3	− 3	− 3	− 6	0.94	− 3
Average	− 4	1	− 3	− 2	− 2	− 2	− 3	− 2	− 1	− 4	2	− 2
**Suburban Riyadh**												
Max	− 3	0	− 2	− 2	0	− 2	− 2	− 2	− 1	− 4	2.35	− 2
Min	− 5	0	− 4	− 4	− 3	− 3	− 3	− 3	− 2	− 6	1.75	− 3
Average	− 4	0	− 3	− 2	− 2	− 3	− 3	− 2	− 1	− 5	2	− 2
**Urban Mahad AD’Dahab**												
Max	− 3	2	− 2	− 2	2	− 2	− 2	− 1	1	− 3	3	− 0.5
Min	− 3	− 2	− 2	− 3	0	− 2	− 2	− 2	− 1	− 4	1	− 2
Average	− 3	0	− 2	− 2	1	− 2	− 2	− 2	0	− 3	2.73	− 1
**Suburban Mahad AD’Dahab**												
Max	− 3	− 1	− 2	− 2	0	− 1	− 1	− 1	− 1	− 3	3	− 1
Min	− 4	− 2	− 4	− 4	− 3	− 3	− 3	− 3	− 3	− 4	1	− 3
Average	− 4	− 2	− 3	− 3	− 2	− 2	− 2	− 2	− 2	− 4	2.09	− 2

**Table 5:** The calculated data of the enrichment factor (EF) for heavy metals of soil and dust samples.

Soil	Al	Cd	Co	Cr	Cu	Mn	Ni	Pb	Ti	Zn
**Urban Riyadh**										
Max	1.53	4.91	0.00	0.80	0.61	1.08	1.22	5.83	0.27	5.02
Min	0.56	0.00	0.00	0.14	0.00	0.86	0.00	1.48	0.08	1.05
Average	0.70	0.57	0.00	0.44	0.07	0.98	0.35	3.79	0.20	1.90
**Suburban Riyadh**										
Max	0.54	0.00	0.29	0.31	0.20	0.96	0.51	3.88	0.27	1.27
Min	0.50	0.00	0.00	0.16	0.00	0.92	0.43	1.64	0.18	0.59
Average	0.52	0.00	0.15	0.24	0.10	0.94	0.47	2.76	0.22	0.93
**Urban Mahad AD’Dahab**										
Max	0.74	6.66	0.05	0.31	1.58	1.47	0.48	4.68	0.45	3.80
Min	0.60	0.00	0.00	0.19	0.28	1.19	0.11	1.67	0.35	1.12
Average	0.67	3.33	0.02	0.25	0.93	1.33	0.30	3.18	0.40	2.46
**Suburban Mahad AD’Dahab**										
Max	0.55	0.00	0.00	0.22	0.86	1.47	0.38	1.37	0.57	1.68
Min	0.52	0.00	0.00	0.15	0.49	1.29	0.19	1.36	0.52	1.06
Average	0.54	0.00	0.00	0.18	0.68	1.38	0.28	1.37	0.55	1.37
**Dust**										
**Urban Riyadh**										
Max	0.68	24.3	0.82	0.69	3.84	1.04	1.23	10.48	0.31	110.8
Min	0.40	0.00	0.06	0.50	0.67	0.81	0.82	1.33	0.11	4.96
Average	0.51	1.97	0.44	0.59	1.38	0.92	1.05	4.06	0.23	29.20
**Suburban Riyadh**										
Max	0.59	0.00	0.46	0.64	0.89	0.95	1.19	4.22	0.28	59.00
Min	0.40	0.00	0.00	0.38	0.41	0.77	0.70	1.44	0.14	8.31
Average	0.51	0.00	0.30	0.53	0.65	0.88	0.97	2.38	0.22	32.20
**Urban Mahad AD’Dahab**										
Max	0.54	23.9	0.64	0.49	9.01	1.24	1.00	13.50	0.43	61.00
Min	0.41	1.38	0.42	0.37	2.21	0.98	0.67	2.96	0.30	15.30
Average	0.48	9.45	0.58	0.41	4.98	1.10	0.81	6.67	0.37	33.60
**Suburban Mahad AD’Dahab**										
Max	0.47	2.73	0.49	0.35	2.33	1.22	0.74	2.84	0.37	38.10
Min	0.40	1.47	0.25	0.24	0.84	1.01	0.52	0.91	0.32	12.00
Average	0.43	2.22	0.39	0.31	1.11	1.09	0.67	1.53	0.34	27.00

**Table 6:** Average the single potential ecological risk (Ei) and integrated potential ecological risk (IR) in soil and dust of urban and suburban areas in Riyadh and Mahad AD’Dahab.

Soil	Ei						RI
	Cd	Cr	Cu	Ni	Pb	Zn	
**Urban Riyadh**							
Max	15	0.3	0.3	1.2	3.9	0.4	18
Min	0.0	0.0	0.0	0.0	0.4	0.1	0
Average	1.7	0.1	0.1	0.2	1.8	0.2	4
**Suburban Riyadh**							
Max	0.0	0.1	0.2	0.5	1.5	0.2	3
Min	0.0	0.0	0.0	0.2	1.4	0.0	2
Average	0.0	0.1	0.1	0.3	1.5	0.1	2
**Urban Mahad AD’Dahab**							
Max	59.0	0.2	2.3	0.7	6.9	1.1	70
Min	0.0	0.1	0.3	0.1	1.4	0.2	2
Average	15.0	0.1	1.1	0.4	3.0	0.5	36
**Suburban Mahad AD’Dahab**							
Max	59.0	0.2	2.3	0.7	6.9	1.1	4
Min	0.0	0.1	0.3	0.1	1.4	0.2	2
Average	15.0	0.1	1.1	0.4	3.0	0.5	3
**Dust**							
**Urban Riyadh**							
Max	156.0	0.6	4.4	2.6	13.2	37.8	177
Min	0.0	0.2	0.8	0.8	1.6	1.3	5.9
Average	14.7	0.3	1.7	1.3	5.0	7.5	30.5
**Suburban Riyadh**							
Max	0.0	0.4	1.1	1.4	5.3	14.4	20.5
Min	0.0	0.1	0.5	0.5	1.7	1.3	4.5
Average	0.0	0.2	0.7	1.1	2.7	7.5	12.3
**Urban Mahad AD’Dahab**							
Max	274.0	0.4	14.8	1.8	24.6	19.6	319
Min	14.8	0.2	3.9	1.2	4.9	5.0	30.9
Average	104.0	0.3	9.1	1.5	12.1	12.3	139
**Suburban Mahad AD’Dahab**							
Max	24.9	0.4	4.0	1.9	4.9	16.7	50.7
Min	14.7	0.1	0.8	0.5	0.8	2.8	19.8
Average	18.6	0.2	1.7	1.0	2.3	8.3	32.1

The IPI values in soil samples collected in the Riyadh and Mahad AD’Dahab areas were < 1, indicating a low level of pollution with HMs. However, based on the maximal values of IPI in samples collected from urban areas of Riyadh and Mahad AD’Dahab, the dust samples were moderately and highly polluted, respectively.

Table 4 shows the values of the geo-accumulation index of the soil and dust samples collected at the Riyadh and Mahad AD’Dahab sites. The results indicate that all soil and dust samples were unpolluted, with the exception of the presence of Zn in the dust samples. In the urban Riyadh area, the Igeo values of Zn ranged from 0.94 to 3.53, suggesting moderate or strong pollution levels. However, the Igeo values of Zn in the dust samples collected at the two sites of the Riyadh suburban area ranged from 1.75 (S18) to 2.35 (S19), suggesting moderate or moderate to strong pollution levels, respectively. In the Mahad AD’Dahab area, the average Igeo values of Zn were 2.73 (urban sites) and 2.09 (suburban sites), indicating moderate to strong pollution levels. Shabbaj et al.^51^ also found that the Igeo value indicated moderate to heavy contamination with Pb and Zn, and heavy to extreme contamination with Cd in urban dust collected in Jeddah, Saudi Arabia.

Table 5 shows the EF values corresponding to the soil and dust samples collected at the Riyadh and Mahad AD’Dahab sites. Among all metals, the highest EF values were recorded for Cd (6.66), Pb (4.68), and Zn (3.80) in soil samples collected at site 3 of the urban Mahad AD’Dahab area, indicating significant enrichment for Cd and moderated enrichment for Zn and Pb. Our findings suggest that site 3 of the urban Mahad AD’Dahab area is a site-specific source of pollution and that the mining operations affect the surrounding area of Mahad AD’Dahab more than does the industrial activity of the Riyadh area. It was previously reported that the soil in areas surrounding the Mahad AD’Dahab mine contains high levels of HMs, such as Cd, Cu, Zn, and Pb^30^. According to the obtained results, the overall urban and suburban dust samples collected in the Riyadh area during the seasons were characterized by deficiency to minimal enrichment for most investigated metals (especially for Al, Ti, Mn, Ni, Co, and Cr). However, for Cd, some urban sites were characterized by significant enrichment (sites 2 and/or 3 in the summer, winter, and spring seasons) or very high enrichment (sites 2 and 3 in the autumn season). In addition, dust samples collected at site 17 in summer, autumn, and winter, as well as at site 15 in winter, were characterized by significant enrichment for Cu. Our results also showed that most investigated sites were characterized by moderate enrichments (EF = 2–5) for HMs. Among all metals, the urban and suburban sites exhibited highest enrichment for Zn, with significant, very high, or extreme enrichment.

**Figure 1:**
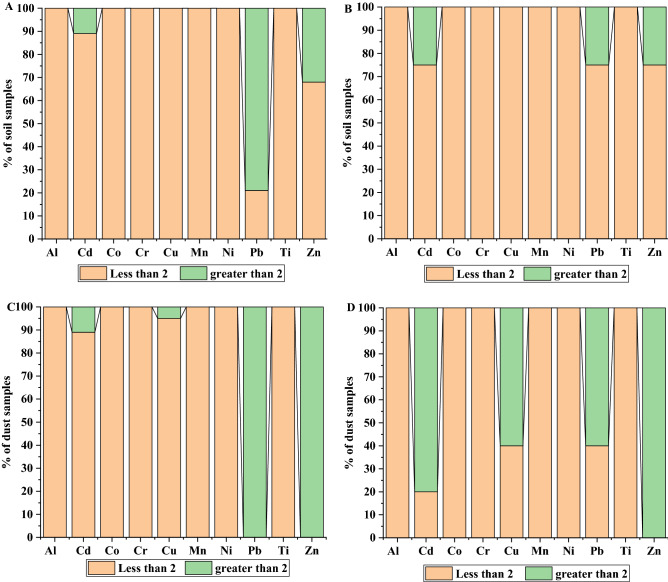
The average percentage of soil and dust samples having EF more or less than 2 [(**A**) soil Riyadh; (**B**) soil Mahad AD’Dahab; (**C**) dust Riyadh; (**D**) dust Mahad AD’Dahab].

The dust samples collected at the suburban mined area of Mahad AD’Dahab had minimal to deficiency or moderate enrichment for all investigated metals (with the exception of Zn, with an EF ranging from 12.0 to 38.1, which indicates very high enrichment). Based on EF values, it was generally observed that urban dust samples were polluted with metals to a greater extent than were suburban dust samples (especially for Cd, Cu, Pb, and Zn). Previously, it was suggested that the EF index could be applied to the determination of the origin of HM contamination, with EF values < 2 indicating that the HM originates from natural sources and EF values > 2 indicating that the HM originates from anthropogenic activities. In this context, the EF values obtained for Al, Co, Cr, Mn, Ni, and Ti at all sites of Riyadh and Mahad AD’Dahab were < 2; therefore, these metals cannot be considered as a major contamination concern. However, the EF values obtained for Cd, Cu, Pb, and Zn were > 2 (Fig. 1), indicating a major contamination concern and the anthropogenic effects of these metals. Among all sites, site 3 at the mined urban area of Mahad AD’Dahab showed the highest EF values for these metals (especially for Pb, Cu, Zn, and Cd), mainly because of its closeness to the gold mine. These results with variations in the calculated coefficients (EF and PI) suggested that anthropogenic activities contributed in the accumulation of HMs in dust and soil of the studied areas. Similar results were reported by Cai et al.^55^ for the contamination of agricultural soils with Pb, Zn, Cd and Hg.

The assessment of the ecological risk potential was also investigated in the current study regarding Cd, Pb, Cu, Cr, Ni, and Zn. Table 6 shows the single potential ecological risk (Ei) and the integrated potential ecological risk (RI). Among all investigated HMs, Cd (maximum Ei value, 274) in dust samples collected at urban sites in Mahad AD’Dahab can be categorized as a moderate ecological risk. Conversely, among all urban and suburban areas, the average integrated potential ecological risk (RI) in dust samples collected at urban areas of Mahad AD’Dahab was categorized as moderate risk.

**Figure 2:**
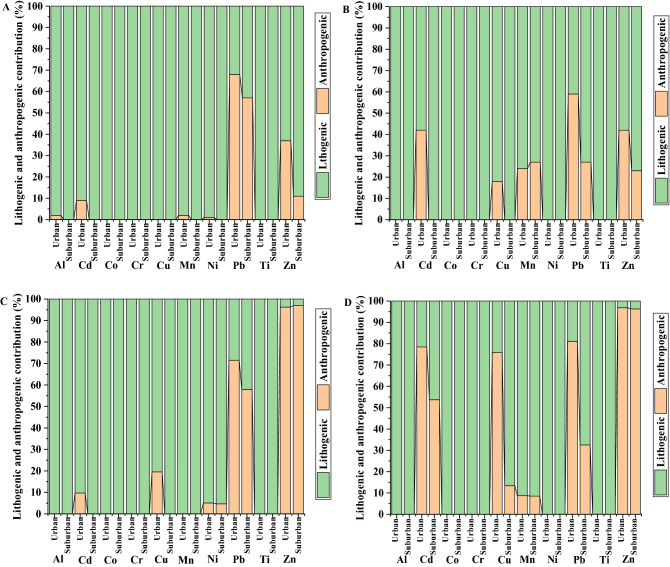
The average percentage of lithogenic and anthropogenic contribution for each metal [(**A**) soil Riyadh; (**B**) soil Mahad AD’Dahab; (**C**) dust Riyadh; (**D**) dust Mahad AD’Dahab].

Figure 2 shows the lithogenic and anthropogenic contribution to the levels of each metal in dust samples. It was reported previously that a value of 50% can be considered as the minimum value for anthropogenic contribution. Therefore, the results obtained indicated that the soil samples had a higher significant anthropogenic contribution (> 50%) was observed for Pb in the urban and suburban area of Riyadh, and urban area of Mahad AD’Dahab. Additionally, the relative higher anthropogenic contributions for Zn (37%) at urban area of Riyadh and for both Cd and Zn (42%) at urban area of Mahad AD’Dahab. Meanwhile, the dust samples had a higher lithogenic contribution (< 50%) regarding Al, Ti, Mn, Ni, Co, and Cr. Conversely, a higher significant anthropogenic contribution (> 50%) was observed for Zn and Pb in the Riyadh area, and for Zn, Pb, Cu and Cd in the Mahad AD’Dahab area. Generally, it can be speculated that, in the studied areas, these metals are originated from anthropogenic sources, indicating the possibility of contamination of the investigated areas with these metals as a result of industrial activities and heavy traffic at Riyadh, or mining activities at Mahad AD’Dahab.

The correlations between HM concentrations in dust samples collected from the Riyadh and Mahad AD’Dahab areas are presented in Supplementary Table S6. Pearson coefficients showed a significant positive correlation among various metals in dust samples. Most of the investigated HMs (with the exception of Cd and Cu at the Riyadh area and Cd, Cu, and Pb at the mining area) were significantly correlated with Fe, Al, or Ti. Our results indicated that Al, Cr, Co, Mn, Ni, Ti, and Zn in dust were more likely to stem from the same sources. However, a significant positive correlation was observed between Cd, Cu, and Pb, suggesting that these HMs in dust samples of the mining area originated from the same sources.

**Figure 3:**
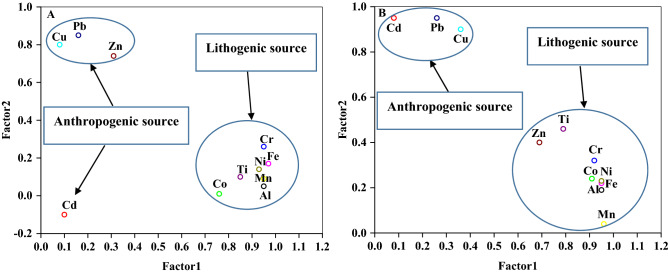
2D plot of the PCA for heavy metals in dust samples of Riyadh (**A**) and Mahad AD’Dahab (**B**).

The results of PCA showed that four components were extracted for HMs in the dust samples at the Riyadh area (affected by industrial activities), whereas two components were extracted for the mining area of Mahad AD’Dahab. In the Riyadh area, Factors 1, 2, 3, and 4 accounted for 57.12%, 15.75%, 11.21%, and 5.98% of the total variance, respectively (Supplementary Table S7). Factor 1 was positively loaded with Al, Cr, Co, Fe, Mn, Ni, and Ti. Factors 2, 3, and 4 were dominated by Cu, Cd, and Zn, respectively. These results suggest that Al, Cr, Co, Fe, Mn, Ni, and Ti may originate from a lithogenic source; in contrast, Cu, Cd, and Zn may stem from anthropogenic sources. Moreover, at the mining area of Mahad AD’Dahab, Factor 1 was dominated by Al, Co, Cr, Fe, Mn, Ni, and Ti, while factor 2 was dominated by Cd, Cu, and Pb. The 2D plot of the PCA suggested that the HMs in both areas came from different sources (Fig. 3). The results of the 2D plot of the PCA showed a high loading for most of the analyzed HMs, including Al, Cr, Fe, Mn, Ni, and Zn, at the Riyadh area, and Al, Co, Cr, Fe, Mn, Ni, Zn, and Ti at the mining area of Mahad AD’Dahab. This suggests that this group of HMs may have a natural origin, because of their correlation with each other and with Fe, Al, or Ti as a reference metal in earth’s crust. A loading between Cd, Cu, and Pb was also observed at the mining area of Mahad AD’Dahab. In addition, there was a loading between Zn, Pb, and Cu at the industrial area of Riyadh. These three metals (Cd, Cu, and Pb) at the mining area, and Zn and Pb in particular at the industrial area of Riyadh, exhibited an EF > 2 in most dust samples, with significant correlation between them. Therefore, these metals may stem from another source (mainly anthropogenic input). A study conducted by Yang et al.^56^ to trace the sources of, and assess the contamination with, HMs in dust samples in a typical mining city in China reported that Ni, Zn, Fe, and Cr were associated with the first factor, which led the authors to suggest that these four metals originated mainly from common sources. However, they found that Cd and Pb were associated with the third factor, suggesting anthropogenic sources^54^. In another study based on EF and multivariate analysis, Almasoud et al.^57^ found that the sources of Zn, Cu, and Pb were primarily anthropogenic, whereas Co, Ni, Fe, and Mn in the soil stemmed from industrial activities. In the mining Mahad AD’Dahab area, Alsaleh et al.^45^ reported that the soil samples were characterized by moderate to high pollution levels of Cd, Cu, Zn, and Pb, whereas the concentrations of Fe, Mn, Ni, Co, and Cr were at background levels.

### Human health risk assessment of HMs in dust and soil in urban and suburban areas

Heavy metals can have a serious impact on human health. For urban areas, the direct risks of HMs in recreational, residential, traffic, and industrial areas generally originate from oral ingestion and dermal exposure^58,59^. In this study, the non-carcinogenic HQ, HI, and ILCR of each HM were computed. The human health risk indicators of dust and soil of < 63 µm for HMs were calculated for all seasons in urban and suburban areas in Riyadh and Mahad AD’Dahab.

#### Non-carcinogenic and carcinogenic hazards assessment of HMs in dust

The HQ values in the urban and suburban areas indicated that the investigated HMs in Mahad AD’Dahab had higher values than those of Riyadh in the exposure pathways for all human categories (except for Cr, Ni, and Pb in suburban areas). In Riyadh and Mahad AD’Dahab, the HQ values of HMs for all dust samples were < 1. Therefore, there were no adverse health effects (Supplementary Tables S8 and S9). Likewise, the HI values in the urban and suburban areas of Riyadh and Mahad AD’Dahab were < 1, thereby suggesting that there was no non-carcinogenic risk (Supplementary Table S10). The HI values indicted that urban dust samples collected from Mahad AD’Dahab had a more serious impact on human health than those from Riyadh. Therefore, more attention should be paid to the HM contamination in dust samples of such residential environments. Li et al.^60^ assessed the health risks of nine HMs (As, Cd, Co, Cr, Pb, Cu, Zn, Mn, and Ni) in road dust in the Bayan Obo Mining Region in Inner Mongolia, China. In their study, the main exposure route was ingestion, and they found that Mn, Cr, Pb, and As were the main contributors to non-cancer risks in both children and adults. Meanwhile, according to the cancer risk assessment, Cr was the main contributor.

The HI values in Riyadh and Mahad AD’Dahab were < 1 for pathway exposure for all human categories. Also, the HI values were in the following order: children up to 6 years > children from 6 to 12 *y* > adults (Supplementary Figs. S5 and S6). In Riyadh, the highest HI values were found in site 16 followed by sites 2 and 3. In Mahad AD’Dahab, the highest HI values were found in site 3 followed by sites 2 and 1. The ΣHI values of each urban and suburban area of Riyadh were < 1 (except for site 16 with a value > 1) (Supplementary Fig. S7). Therefore, it could be speculated that there was no significant risk of non-carcinogenic health effects in this area (except for site 16). Additionally, the ΣHI values in sites 2 and 3 were close to 1 at 9.26E−01 and 8.97E−01, respectively; therefore, non-carcinogenic risks could be possible.

Al-Rashdi et al.^40^ assessed the health risks of HMs (Fe, Pb, Cu, Mn, Zn, Co, and Cd) in dust from the Dammam area. The HQ and HI values were found to be < 1. However, they suggested that the potential health risks for children and adults due to exposure to HMs cannot be ignored. Similarly, Shabbaj et al.^51^ assessed the health risks of HMs (Fe, Mn, Zn, Pb, Cd, V, Co, Ni, As, Cr, and Cu) in dust from the Jeddah area. They found no potential health risks for the investigated HMs (except for As). However, in the current study, the HI values for all the human categories of each urban area of Mahad AD’Dahab were > 1 (except for site 1 = 9.28E−01); thus, the Mahad AD’Dahab sites posed non-carcinogenic risks. Based on the overall average the hazard index (HI), higher non-carcinogenic risks in urban and suburban in dust samples of Mahad AD’Dahab were observed than those of Riyadh (Supplementary Fig. S8). In this context, Al Bakheet et al.^28^ reported that high concentrations of Pb, Cd, and Hg were observed in the blood samples of residents of Mahad AD’Dahab compared with those of Riyadh, and thus affected genes.

The relationships between the characteristics of dust pollution indicators, ecological risk (RI), and human health risks were investigated. The results showed that there was a significant correlation between the ΣHI and dust pollution indicators (IPI, EF, and I_geo_) and RI (Table 8). In Riyadh, the significant relationship (r) between the ΣHI and dust pollution indicators of IPI, average EF, and average I_geo_ had values of 0.77, 0.65, and 0.98, respectively (Table 8). In Mahad AD’Dahab, the r values between ΣHI and dust pollution indicators of IPI and average I_geo_ were 0.89 and 0.97, respectively. Moreover, significant relationship (r = 0.75) was observed between the ΣHI and RI in current study. Therefore, it could be concluded from the results that the characteristics of dust pollution indicators and RI could be used to predict human health risks in such areas.

The lifetime cancer risks of Cr and Pb in dust for children up to 6 y, children from 6 to 12 years, and adults for different exposure pathways were calculated and presented in Table 7. The USEPA recommends a threshold of 1.00E−06^61^. In urban and suburban areas of Riyadh and Mahad AD’Dahab, the ILCRing and ILCRder for Cr were between 1.00E−06 and 1.00E−04 in all categories for human exposure pathways, thereby indicating that the inhabitants around the study areas of Riyadh and Mahad AD’Dahab may have a high risk of lifetime cancer development. On the contrary, the ILCRing and ILCRder for Pb were < 1.00E−06 in all categories for human exposure pathways, thereby indicating a lower risk^61^.

**Table 7:** Average incremental lifetime cancer risk (ILCR) for Cr and Pb of dust in urban and suburban areas in Riyadh and Mahad AD’Dahab.

			Children up to 6 year		Children up to 6–12 year		Adult		Sum	
			Cr	Pb	Cr	Pb	Cr	Pb	Cr	Pb
**Average incremental lifetime cancer risk (ILCR) for Cr and Pb of dust**										
Riyadh	ILCR_ing_	Urban	1.27E−05	1.17E−07	4.20E−06	3.88E−08	8.42E−06	7.76E−08	2.53E−05	2.33E−07
		Suburban	1.04E−05	6.26E−08	3.44E−06	2.07E−08	6.89E−06	4.15E−08	2.07E−05	1.25E−07
Mahad AD'Dahab	ILCR_ing_	Urban	1.28E−05	2.81E−07	4.24E−06	9.32E−08	8.50E−06	1.87E−07	2.55E−05	5.61E−07
		Suburban	7.94E−06	5.39E−08	2.63E−06	1.78E−08	5.26E−06	3.57E−08	1.58E−05	1.07E−07
Riyadh	ILCR_der_	Urban	6.82E−06	8.18E−10	6.77E−06	8.12E−10	9.49E−06	1.14E−09	2.31E−05	2.77E−09
		Suburban	5.58E−06	4.37E−10	5.54E−06	4.34E−10	7.76E−06	6.08E−10	1.89E−05	1.48E−09
Mahad AD'Dahab	ILCR_der_	Urban	6.88E−06	1.96E−09	6.84E−06	1.95E−09	9.58E−06	2.73E−09	2.33E−05	6.64E−09
		Suburban	4.26E−06	3.76E−10	4.23E−06	3.73E−10	5.93E−06	5.24E−10	1.44E−05	1.27E−09
Riyadh	ILCR_ing+der_	Urban	1.95E−05	1.18E−07	1.10E−05	3.96E−08	1.79E−05	7.87E−08	4.84E−05	2.36E−07
		Suburban	1.60E−05	6.30E−08	8.98E−06	2.11E−08	1.47E−05	4.21E−08	3.96E−05	1.26E−07
Mahad AD'Dahab	ILCR_ing+der_	Urban	1.97E−05	2.83E−07	1.11E−05	9.52E−08	1.81E−05	1.90E−07	4.88E−05	5.68E−07
		Suburban	1.22E−05	5.43E−08	6.86E−06	1.82E−08	1.12E−05	3.62E−08	3.03E−05	1.09E−07
**Average incremental lifetime cancer risk (ILCR) for Cr and Pb of soil**										
Riyadh	ILCR_ing_	Urban	1.02E−05	5.64E−08	3.39E−06	1.87E−08	6.79E−06	3.74E−08	2.04E−05	1.13E−07
		Suburban	1.50E−05	6.61E−08	4.97E−06	2.19E−08	9.94E−06	4.38E−08	2.99E−05	1.32E−07
Mahad AD'Dahab	ILCR_ing_	Urban	1.59E−05	2.69E−07	5.28E−06	8.91E−08	1.06E−05	1.78E−07	3.18E−05	5.37E−07
		Suburban	1.50E−05	9.20E−08	4.95E−06	3.04E−08	9.92E−06	6.10E−08	2.98E−05	1.83E−07
Riyadh	ILCR_der_	Urban	5.50E−06	3.94E−10	5.46E−06	3.91E−10	7.65E−06	5.48E−10	1.86E−05	1.33E−09
		Suburban	8.05E−06	4.61E−10	8.00E−06	4.58E−10	1.12E−05	6.42E−10	2.73E−05	1.56E−09
Mahad AD'Dahab	ILCR_der_	Urban	8.56E−06	1.88E−09	8.50E−06	1.87E−09	1.19E−05	2.62E−09	2.90E−05	6.36E−09
		Suburban	8.03E−06	6.42E−10	7.98E−06	6.38E−10	1.12E−05	8.94E−10	2.72E−05	2.17E−09
Riyadh	ILCR_ing+der_	Urban	1.57E−05	5.68E−08	8.85E−06	1.91E−08	1.44E−05	3.80E−08	3.90E−05	1.14E−07
		Suburban	2.31E−05	6.65E−08	1.30E−05	2.23E−08	2.12E−05	4.44E−08	5.72E−05	1.33E−07
Mahad AD'Dahab	ILCR_ing+der_	Urban	2.45E−05	2.71E−07	1.38E−05	9.10E−08	2.25E−05	1.81E−07	6.08E−05	5.43E−07
		Suburban	2.30E−05	9.26E−08	1.29E−05	3.11E−08	2.11E−05	6.19E−08	5.70E−05	1.86E−07

Generally, the mean values of ILCR for Cr and Pb in urban areas of Mahad AD’Dahab were higher than those for Riyadh. On the contrary, the mean values of ILCR for Cr and Pb in suburban areas of Mahad AD’Dahab were lower than those for Riyadh in all human categories. Additionally, among the two metals (Cr and Pb), the ILCR values of Cr in Mahad AD’Dahab and those in Riyadh for all human risk categories were close to the recommended threshold of 1.00E−06 to 1.00E−04, thereby indicating a possible risk of lifetime cancer development in the inhabitants around the study sites^61^.

### Non-carcinogenic and carcinogenic hazards assessment of HMs in soil (< 63 µm)

The HQ and HI values in the soil samples (< 63 µm) of the urban and suburban areas indicated that the investigated HM concentrations in Mahad AD’Dahab were higher than those in Riyadh in the exposure pathways for all human categories (with the exception of Cd and Cr).

In Riyadh and Mahad AD’Dahab, the HQ values of HMs for all soil samples were < 1, thereby suggesting no adverse health impacts (Supplementary Tables S11 and S12). Additionally, the HI values in the urban and suburban areas of Riyadh and Mahad AD’Dahab were < 1 for all human categories, thereby suggesting that there was no non-carcinogenic risk (Supplementary Table S13). The HI values indicated that urban soil samples collected from Mahad AD’Dahab had a more serious impact on human health than those in Riyadh. Therefore, more attention should paid to the HM contamination in soil samples of such residential environments. In Riyadh, the HQ and HI values in urban areas were lower than those in suburban areas (except for Cu and Zn). In Mahad AD’Dahab, the HQ and HI values in urban areas were higher than those in suburban areas (except for Ni). The HI values decreased in the following order: 6 years > children from 6 to 12 years > adults (Supplementary Figs. S9 and S10).

The ΣHI values for all human categories of each site in the urban and suburban areas of Riyadh were < 1 (Supplementary Fig. S11), indicating no adverse non-carcinogenic health effects. However, the ΣHI values for all human categories in the urban and suburban sites in Mahad AD’Dahab were > 1; thus, the Mahad AD’Dahab sites posed non-carcinogenic risks (Supplementary Figs. S12 and S13). Alsaleh^62^ assessed the health risks of HMs (Cd, Cu, Pb, and Zn) in the soil of the Riyadh and Mahad AD’Dahab areas. In their study, the ΣHI values for all human categories in Riyadh and Mahad AD’Dahab areas were 1.07 and 28.90, respectively, which were above the allowable safe dose limits for non-cancer health effects. Mahad AD’Dahab area had higher ΣHI values than those obtained from our study. The higher values of ΣHI obtained by Alsaleh^62^ could be explained by soil samples that were collected from the nearby Mahad AD’Dahab mine.

The relationships between the characteristics of soil pollution indicators, RI, and human health risks were established. The relationships showed that there was a significant correlation between the ΣHI and soil pollution indicators (IPI, EF, and I_geo_) and RI (Table 8). In Riyadh, the r values between ΣHI and the soil pollution indicators of IPI, average EF, and average I_geo_ were 0.69, − 0.59, and 0.95, respectively. In Mahad AD’Dahab, the r values between ΣHI and the soil pollution indicators of IPI, average EF, and average I_geo_ were 0.98, 0.99, and 1.00, respectively. In addition, there was a significant relationship (r = 0.98) between the ΣHI and RI. From the obtained results, it could be concluded that the characteristics of soil pollution indicators and RI could be used to predict human health risks in such areas^63^.

**Table 8:** Relationship between the characteristics of dust, soil pollution and the human health risks.

Indices	Riyadh				
	∑HI	RI^+^	IPI	EF	I_geo_
**Dust samples**					
∑HI	1.00				
RI^+^	0.10	1.00			
IPI	0.77*	− 0.01	1.00		
EF^+^	0.65*	0.08	0.94*	1.00	
I_geo_^+^	0.98*	0.07	0.77*	0.66*	1.00
**Mahad AD'Dahab**					
∑HI	1.00				
RI^+^	0.75	1.00			
IPI	0.89*	0.94*	1.00		
EF^+^	0.57	0.75	0.65	1.00	
I_geo_^+^	0.97*	0.84	0.96*	0.54	1.00
**Soil (less than 63 µm)**					
∑HI	1.00				
RI^+^	− 0.21	1.00			
IPI	0.69*	0.32	1.00		
EF^+^	− 0.59*	0.74*	0.09	1.00	
I_geo_^+^	0.95*	− 0.20	0.79*	− 0.50*	1.00
**Mahad AD'Dahab**					
∑HI	1.00				
RI^+^	0.98*	1.00			
IPI	0.98*	1.00*	1.00		
EF^+^	0.99*	1.00*	1.00*	1.00	
I_geo_^+^	1.00*	0.99*	0.99*	0.99*	1.00

*Significant.

The lifetime cancer risks due to Cr and Pb in soil for all the pathways for children up to 6 years, children from 6 to 12 years, and adults were calculated in this study. Table 7 shows the average ILCR for Cr and Pb in soil in the urban and suburban areas of Riyadh and Mahad AD’Dahab. In the urban and suburban areas of Riyadh and Mahad AD’Dahab, the ILCR_ing_, ILCRder, and ILCR_ing+der_ were between 1.00E−06 and 1.00E−04 in all human categories, thereby indicating that the inhabitants around the study areas of Riyadh and Mahad AD’Dahab may have a high risk of lifetime cancer development. On the contrary, the ILCRing, ILCRder, and ILCR_ing+der_ for Pb were < 1.00E−06 in all human categories, thereby indicating lower risk^61^. Generally, the mean values of ILCR for Cr and Pb in urban and suburban areas of Mahad AD’Dahab were higher than those in Riyadh (except for Cr in suburban areas). Also, among the two metals (Cr and Pb), the ILCR values of Cr in Mahad AD’Dahab and Riyadh for all human categories were close to the recommended threshold of 1.00E−06 to 1.00E−04, thereby suggesting a possible risk to the inhabitants around the study sites^61^. Previously, it has been shown that ingestion was the most hazardous pathway for developing cancer via HMs intake, followed by dermal contact and inhalation. Moreover, it was observed that carcinogenic risks for children via ingestion and dermal contact were more than that of adults^64^.

## Conclusions

Levels and sources of HMs present in dust and soil of urban and suburban areas in Riyadh and Mahad AD’Dahab cities were studied and associated ecological and human health risks were estimated in this study. Overall, the levels of studied HMs were higher in dust samples than that of soil samples collected from the studied areas. The enrichment factor and PCA analyses exhibited that Al, Co, Cr, Fe, Mn, Ni, Ti, and Zn were of lithogenic origin, whereas, Cu, Zn, and Pb in Riyadh and Cd, Cu, Pb, and Zn in the Mahad AD’Dahab were of anthropogenic activities. The HI values for dust and soil (< 63 µm) samples were as: children up to 6 years > children from 6 to 12 years > adults. The HI values for all human categories for dust in urban areas of Mahad AD’Dahab and soil samples (< 63 µm) in urban and suburban areas of Mahad AD’Dahab were > 1, indicating non-carcinogenic risk. The average values of ILCR for dust and soil (< 63 µm) samples from both urban and suburban areas of Riyadh and Mahad AD’Dahab were close to the recommended threshold for Cr; however, were lower for Pb. Higher levels and ecological and human health risks of HMs in residential areas of Riyadh and Mahad AD’Dahab could be owing to industrial and mining activities in these cities. Therefore, ecological and human health risk assessment showed that soil and dust of Mahad AD’Dahab were more polluted as compared to Riyadh. It is therefore recommended to monitor HMs pollution in dust and soil particles of such residential environments for sustainable ecosystem and human health.

## Supplementary Information

Supplementary Information.

## Data Availability

All data are fully available without restriction.

## References

[1] Wong CS, Li X, Thornton I (2006). Urban environmental geochemistry of trace metals. Environ. Pollut..

[2] Wang Y, Duan X, Wang L (2020). Spatial distribution and source analysis of heavy metals in soils influenced by industrial enterprise distribution: Case study in Jiangsu Province. Sci. Total. Environ..

[3] Al-Sareji OJ, Grmasha RA, Salman JM, Hashim KS (2021). Street dust contamination by heavy metals in babylon governorate, Iraq. J. Eng. Sci. Technol..

[4] Alloway, B. J. Sources of heavy metals and metalloids in soils. In* Heavy Metals Soils.* 11–50 (2013).

[5] Luo X-S, Ding J, Xu B, Wang Y-J, Li H-B, Yu S (2012). Incorporating bioaccessibility into human health risk assessments of heavy metals in urban park soils. Sci. Total. Environ..

[6] Lee CSL, Li X, Shi W, Cheung SCN, Thornton I (2006). Metal contamination in urban, suburban, and country park soils of Hong Kong: A study based on GIS and multivariate statistics. Sci. Total Environ..

[7] El-Sergany MM, El-Sharkawy MF (2011). Heavy metal contamination of airborne dust in the environment of two main cities in the Eastern Province of Saudi Arabia. JKAU. Met. Environ. Arid. Land. Agric. Sci..

[8] Varoujan, K. S., Nadhir, A.-A. & Sven, K. Sand and dust storm events in Iraq. *Nat. Sci.* **5**(10), 1084–1094 (2013).

[9] Lee S-W, Lee B-T, Kim J-Y, Kim K-W, Lee J-S (2006). Human risk assessment for heavy metals and as contamination in the abandoned metal mine areas. Korea. Environ. Monit. Assess..

[10] Alotaibi MO, Albedair LA, Alotaibi NM, Elobeid MM, Al-Swadi HA, Alasmary Z, Ahmad M (2022). Pollution indexing and health risk assessment of heavy-metals-laden indoor and outdoor dust in elementary school environments in Riyadh, Saudi Arabia. Atmosphere.

[11] Kampa M, Castanas E (2008). Human health effects of air pollution. Environ. Pollut..

[12] Roussel H, Waterlot C, Pelfrêne A, Pruvot C, Mazzuca M, Douay F (2010). Cd, Pb and Zn oral bioaccessibility of urban soils contaminated in the past by atmospheric emissions from two lead and zinc smelters. Arch. Environ. Contam. Toxicol..

[13] Madrid F, Biasioli M, Ajmone-Marsan F (2008). Availability and bioaccessibility of metals in fine particles of some urban soils. Arch Environ. Contam. Toxicol..

[14] Voutsa D, Samara C (2002). Labile and bioaccessible fractions of heavy metals in the airborne particulate matter from urban and industrial areas. Atmos. Environ..

[15] Abrahams PW (2002). Soils: Their implications to human health. Sci. Total. Environ..

[16] USEPA, N. *Child-Specific Exposure Factors Handbook*. (EPA, 2008).

[17] Johnson DL, Bretsch JK (2002). Soil lead and children’s blood lead levels in Syracuse, NY, USA. Environ. Geochem. Health..

[18] Yamamoto N, Takahashi Y, Yoshinaga J, Tanaka A, Shibata Y (2006). Size distributions of soil particles adhered to children’s hands. Arch. Environ. Contam. Toxicol..

[19] Irshad S, Liu G, Yousaf B, Ullah H, Ali MU, Rinklebe J (2019). Estimating the pollution characteristics and health risks of potentially toxic metal (loid) s in urban-industrial soils in the Indus basin, Pakistan. Environ. Monit. Assess..

[20] Rodrigues, S. M. & Römkens, P. F. Human health risks and soil pollution. In* Soil Pollution* 217–250 (2018).

[21] Al Jassir M, Shaker A, Khaliq M (2005). Deposition of heavy metals on green leafy vegerables sold on roadsides of Riyadh City, Saudi Arabia. Bull. Environ. Contam. Toxicol..

[22] Al-Rajhi MA, Seaward MRD, Al-Aamer AS (1996). Metal levels in indoor and outdoor dust in Riyadh, Saudi Arabia. Environ. Int..

[23] El-Desoky GE, Aboul-Soud MA, Al-Othman ZA, Habila M, Giesy JP (2014). Seasonal concentrations of lead in outdoor and indoor dust and blood of children in Riyadh, Saudi Arabia. Environ. Geochem. Health..

[24] Al-Farraj A, Al-Wabel M (2007). Evaluation of soil pollution around mahad ad’dahab mine. J. Saudi. Soc. Agric. Sci..

[25] Al-Farraj A, Al-Wabel M (2007). Heavy metals accumulation of some plant species grown. J. Appl. Sci..

[26] Al-Farraj A, Al-Otabi T, Al-Wabel M (2009). Accumulation coefficient and translocation factor of heavy metals through *Ochradenus baccatus* plant grown on mining area at Mahad AD'Dahab, Saudi Arabia. WIT. Trans. Ecol. Environ..

[27] Al-Farraj AS, Usman AR, Al-Otaibi SH (2013). Assessment of heavy metals contamination in soils surrounding a gold mine: Comparison of two digestion methods. Chem. Ecol..

[28] Al Bakheet SA, Attafi IM, Maayah ZH, Abd-Allah AR, Asiri YA, Korashy HM (2013). Effect of long-term human exposure to environmental heavy metals on the expression of detoxification and DNA repair genes. Environ. Pollut..

[29] USEPA. Microwave assisted acid digestion of sediments, sludges, soils, and oils. *SW-846 method A, 3051* (1994).

[30] Chen T-B (2005). Assessment of heavy metal pollution in surface soils of urban parks in Beijing, China. Chemosphere.

[31] Faiz Y, Tufail M, Javed MT, Chaudhry MM (2009). Road dust pollution of Cd, Cu, Ni, Pb and Zn along Islamabad expressway, Pakistan. Microchem. J..

[32] Wang L, Lu X, Ren C, Li X, Chen C (2014). Contamination assessment and health risk of heavy metals in dust from Changqing industrial park of Baoji, NW China. Environ. Earth. Sci..

[33] Turekian KK, Wedepohl KH (1961). Distribution of the elements in some major units of the earth's crust. Geol. Soc. Am. Bull..

[34] Hakanson L (1980). An ecological risk index for aquatic pollution control. A sedimentological approach. Water. Res..

[35] Yisa J, Jacob JO, Onoyima CC (2012). Assessment of toxic levels of some heavy metals in road deposited sediments in Suleja, Nigeria. Am. J. Chem..

[36] Sutherland R, Tack F, Tolosa C, Verloo M (2000). Operationally defined metal fractions in road deposited sediment, Honolulu, Hawaii. J. Environ. Qual..

[37] Hernandez L, Probst A, Probst JL, Ulrich E (2003). Heavy metal distribution in some French forest soils: Evidence for atmospheric contamination. Sci. Total. Environ..

[38] Olubunmi FE, Olorunsola OE (2010). Evaluation of the status of heavy metal pollution of sediment of Agbabu bitumen deposit area, Nigeria. Eur. J. Sci. Res..

[39] Yu S, Li X-D (2011). Distribution, availability, and sources of trace metals in different particle size fractions of urban soils in Hong Kong: Implications for assessing the risk to human health. Environ. Pollut..

[40] Acosta JA, Cano AF, Arocena JM, Debela F, Martínez-Martínez S (2009). Distribution of metals in soil particle size fractions and its implication to risk assessment of playgrounds in Murcia City (Spain). Geoderma.

[41] USEPA. Integrated Risk Information System (2011) Environmental Protection Agency Region I, Washington, DC, USA (2019).

[42] USEPA. *Risk Assessment Guidance for Superfund: Volume III—Part A, Process for Conducting Probabilistic Risk Assessment, 20460*. (US Environmental Protection Agency, Office of Emergency and Remedial, 2001).

[43] Yutong Z, Qing X, Shenggao L (2016). Distribution, bioavailability, and leachability of heavy metals in soil particle size fractions of urban soils (northeastern China). Environ. Sci. Pollut. Res..

[44] US Environmental Protection Agency (USEPA). Integrated Risk Information System of the US Environmental Protection Agency (2012).

[45] Alsaleh KA, Meuser H, Usman AR, Al-Wabel MI, Al-Farraj AS (2018). A comparison of two digestion methods for assessing heavy metals level in urban soils influenced by mining and industrial activities. J. Environ. Manag..

[46] Jian-Min Z, Zhi D, Mei-Fang C, Cong-Qiang L (2007). Soil heavy metal pollution around the Dabaoshan mine, Guangdong province, China. Pedosphere.

[47] Al-Rashdi A, Ebqa'ai M, Harb M, Faidi F (2017). A solid phase extraction procedure for the determination of heavy metals in street dust from Dammam, Kingdom of Saudi Arabia and Estimation of the health risk. J. Mater. Environ. Sci..

[48] Yao Q, Wang X, Jian H, Chen H, Yu Z (2015). Characterization of the particle size fraction associated with heavy metals in suspended sediments of the Yellow River. Int. J. Environ. Res..

[49] Hardy M, Cornu S (2006). Location of natural trace elements in silty soils using particle-size fractionation. Geoderma.

[50] Lindsay, W. Phosphorus. In *Chemical Equilibria in Soils*. (Wiley Inter science, 1979).

[51] Shabbaj, I. I., Alghamdi, M. A., Shamy, M., Hassan, S. K., Alsharif, M. M. & Khoder, M. I. Pollution assessment and health risk implication of human exposure to heavy metals in road dusts from different functional areas in Jeddah, Saudi Arabia (2017). 10.3390/ijerph15010036 PMC5799873 29278373

[52] Jiang HH, Cai LM, Hu GC, Wen HH, Luo J, Xu HQ, Chen LG (2021). An integrated exploration on health risk assessment quantification of potentially hazardous elements in soils from the perspective of sources. Ecotoxicol. Environ. Saf..

[53] Wang HZ, Cai LM, Wang QS, Hu GC, Chen LG (2021). A comprehensive exploration of risk assessment and source quantification of potentially toxic elements in road dust: A case study from a large Cu smelter in central China. CATENA.

[54] Cai LM, Wang QS, Wen HH, Luo J, Wang S (2019). Heavy metals in agricultural soils from a typical township in Guangdong Province, China: Occurrences and spatial distribution. Ecotoxicol. Environ. Saf..

[55] Cai L, Xu Z, Ren M, Guo Q, Hu X, Hu G, Wan H, Peng P (2012). Source identification of eight hazardous heavy metals in agricultural soils of Huizhou, Guangdong Province, China. Ecotoxicol. Environ. Saf..

[56] Yang Y, Chen F, Zhang L, Liu J, Wu S, Kang M (2012). Comprehensive assessment of heavy metal contamination in sediment of the Pearl River Estuary and adjacent shelf. Mar. Pollut. Bull..

[57] Almasoud FI, Usman AR, Al-Farraj AS (2015). Heavy metals in the soils of the Arabian Gulf coast affected by industrial activities: Analysis and assessment using enrichment factor and multivariate analysis. Arab. J. Geosci..

[58] De Miguel E, Iribarren I, Chacon E, Ordonez A, Charlesworth S (2007). Risk-based evaluation of the exposure of children to trace elements in playgrounds in Madrid (Spain). Chemosphere.

[59] Zheng N, Liu J, Wang Q, Liang Z (2010). Health risk assessment of heavy metal exposure to street dust in the zinc smelting district, Northeast of China. Sci. Total. Environ..

[60] Li K, Liang T, Wang L, Yang Z (2015). Contamination and health risk assessment of heavy metals in road dust in Bayan Obo Mining Region in Inner Mongolia, North China. J. Geogr. Sci..

[61] USEPA. Exposure and human health reassessment of 2, 3, 7, 8‐tetrachlorodibenzo‐p‐dioxin (TCDD) and related compounds National Academy Sciences (NAS) review draft. National Center for Environmental Assessment, US EPA Washington, DC, http://cutt.us/W0CGV (2004), Accessed 20 Sept 2021.

[62] Alsaleh KA (2014). Comparative Study for Assessment of Some Chemical Extractants to Determine Total Content and Forms of Heavy Metals in Soil.

[63] Yari AA, Varvani J, Zare R (2021). Assessment and zoning of environmental hazard of heavy metals using the Nemerow integrated pollution index in the vineyards of Malayer city. Acta Geophys..

[64] Gabarrón M, Faz A, Acosta JA (2017). Soil or dust for health risk assessment studies in urban environment. Arch. Environ. Contam. Toxicol..

[65] Alharbi BH, Pasha MJ, Alotaibi MD, Alduwais AK, Al-Shamsi MAS (2020). Contamination and risk levels of metals associated with urban street dust in Riyadh, Saudi Arabia. Environ. Sci. Pollut. Res..

[66] Idris AM, Alqahtani FM, Said TO, Fawy KF (2020). Contamination level and risk assessment of heavy metal deposited in street dusts in Khamees-Mushait city, Saudi Arabia. Hum. Ecol. Risk. Assess..

[67] Shabbaj II, Alghamdi MA, Shamy M, Hassan SK, Alsharif MM, Khoder MI (2018). Risk assessment and implication of human exposure to road dust heavy metals in Jeddah, Saudi Arabia. Int. J. Environ. Res..

[68] Kadhum SA (2020). A preliminary study of heavy metals pollution in the sandy dust storms and its human risk assessment from middle and south of Iraq. Environ. Sci. Pollut. Res..

[69] Doabi SA, Karami M, Afyuni M, Yeganeh M (2018). Pollution and health risk assessment of heavy metals in agricultural soil, atmospheric dust and major food crops in Kermanshah province, Iran. Ecotoxicol. Environ. Saf..

[70] Aminiyan MM, Baalousha M, Mousavi R, Aminiyan FM, Hosseini H, Heydariyan A (2018). The ecological risk, source identification, and pollution assessment of heavy metals in road dust: A case study in Rafsanjan, SE Iran. Environ. Sci. Pollu. Res..

[71] Cao Z, Yang Y, Lu J, Zhang C (2011). Atmospheric particle characterization, distribution, and deposition in Xi’an, Shaanxi Province, Central China. Environ. Pollut..

[72] Yeung ZLL, Kwok RCW, Yu KN (2003). Determination of multi-element profiles of street dust using energy dispersive X-ray fluorescence (EDXRF). Appl. Radiat. Isot..

[73] De Miguel E, Llamas JF, Chacón E, Berg T, Larssen S, Røyset O, Vadset M (1997). Origin and patterns of distribution of trace elements in street dust: Unleaded petrol and urban lead. Atmos. Environ..

[74] Ferreira-Baptista L, De Miguel E (2005). Geochemistry and risk assessment of street dust in Luanda, Angola: A tropical urban environment. Atmos. Environ..

[75] Huang S (2009). Multivariate analysis of trace element concentrations in atmospheric deposition in the Yangtze River Delta, East China. Atmos. Environ..

[76] VROM. The new Dutch List. Intervention values and target values: soil quality standards. The Hague, the Netherlands: The Netherlands Ministry of Housing, Spatial Planning and Environment, Department of Soil Protection. Retrieved from http://www.axys.cz/doc/en/Dutchlist.pdf (2001), Accessed 10 Mar 2022.

