# Supplementary material for: Sources, toxicity potential, and human health risk assessment of heavy metals-laden soil and dust of urban and suburban areas as affected by industrial and mining activities
Source: Sci Rep. 2022 May 28;12:8972. doi: 10.1038/s41598-022-12345-8 (PMC9148304; doi:10.1038/s41598-022-12345-8)
Supplement: Supplementary file 1 — Supplementary Information. [file 41598_2022_12345_MOESM1_ESM.docx]

**Supplementary data**

**Sources, toxicity potential, and human health risk assessment of heavy metals-laden soil and dust of urban and suburban areas as affected by industrial and mining activities**

Hamed A. Al-Swadi^1,2*^; Abdullah S. Al-Farraj^1^; Mohammad I. Al-Wabel^1^; Abdulelah Al-Faraj^3^; Adel R. A. Usman^1,4^; Munir Ahmed^1^

^1^Soil Science Department, King Saud University, PO Box: 2460; Riyadh: 11451, Saudi Arabia

^2^Department of Soil, Water and Environment, Faculty of Agriculture, Food and Environment, Sana'a University, Yemen

^3^Agriculture Engineering Department, College of Food and Agriculture Sciences, King Saud University, Riyadh, Saudi Arabia

^4^Department of Soils and Water, Faculty of Agriculture, Assiut University, Assiut 71526, Egypt

***Corresponding authors.** E-mail: halswadi[@ksu.edu.sa](mailto:@ksu.edu.sa); [h.alswadi@hotmail.com](mailto:h.alswadi@hotmail.com);

Tel.: +966541671566

**Table S1** Definitions of the parameters and values of the variables for the human health risk assessment.

| Variable | Unit | Definition | Value | References |
| --- | --- | --- | --- | --- |
| ADD _ingestion_  ADD _dermal_ | mg kg^-1^ day^-1^ | The average daily dose contacted through ingestion and dermal |  | In this study |
| C_dust_ | mg kg^-1^ | Concentration of metal in dust |  | In this study |
| IR_ingestion_ | mg day^-1^ | The ingestion rate of dust | 200 for children up to 6 years, 100 for children 6-12 years and 50 for adult | [52] |
| EF | day | The exposure frequency | 350 | [35] |
| ED | year | The exposure duration | 5 years for children up to 6 years, 6 years for children up to 6-12 years and 58 for adult | [52] |
| F | unitless | Factor= 10^-6^ |  | [35] |
| ABS | unitless | the dermal absorption factor | 0.03 for AS and 0.001 for other metals | [5,35,53] |
| BW | kg | average body weight | 16 kg for children up to 6 years, 29 kg from 6 to 12 years and 70 for adult | [52] |
| AT | day | average lifetime | For non-carcinogenic= ED*365  5*365 for children up to 6 years, 5*365 for children 6­­-12 years and 58*365 for adult.  For carcinogenic  70*365 for three age categories | [52] |
| SA | cm^2^ | the exposed skin surface area | 6980 for children up to 6 years, 10470 for children up to 6-12 years and 18150 for adult | [52] |
| RFD_ingestion_ | mg kg^-1^ day^-1^ | Chronic oral reference dose | Cd= 0.001; Co= 3.00E-04; Cr=3.00E-03; Cu= 4.00E-02; Mn= 1.40E-02; Ni= 2.00E-02; Pb= 3.50E-03; Zn= 3.00E-01 | [54] |
| RFD_dermal_ | mg kg^-1^ day^-1^ | Chronic dermal reference dose | Co= 1.60E-02; Cr=6.00E-05; Cu= 1.20E-02; Cd= 2.50E-05 (Luo et al., 2012); Mn=1.84E-03; Ni= 5.40E-03; Pb= 3.25E-04; Zn= 6.00E-02 | [35,54] |
| SF_ing_ |  | Cancer slope factor | Cr= 5.00E-01; Pb= 8.5E-03 | [5,54] |
| SF_der_ |  | Cancer slope factor | Cr= 6.50E-03; Pb= 8.50E-03 | [5,35,54] |

**Table S2** The recovery of heavy metals content in the three certificated reference material (Till 1, Till 2 and Till 4) digested by EPA 3051 method

|  | Al | Cd | Co | Cr | Cu | Fe | Mn | Ni | Pb | Ti | Zn |
| --- | --- | --- | --- | --- | --- | --- | --- | --- | --- | --- | --- |
|  | **%** | | | | | | | | | | |
| Till 1 | 76.4 | - | 61.3 | 56.8 | 69.2 | 57.4 | 73.5 | 55.6 | - | - | 72.7 |
| Till 2 | 83.7 | - | 52.1 | 75.5 | 65.2 | 62.5 | 74.3 | 64.8 | 49.8 | - | 74.6 |
| Till 4 | 71.5 | - | 49.9 | 66.1 | 53.1 | 57.8 | 65.3 | 72.3 | 61.5 | - | 76.1 |
| Average | 77.2 | - | 54.4 | 66.1 | 62.5 | 59.3 | 71.0 | 64.2 | 55.7 | - | 74.5 |

**Table S3a** Minimum, maximum and average concentrations of the heavy metals (mg kg^-1^) in the different particle size fractions of urban and suburban soils at industrial impacted area of Riyadh.

| Soil fraction µm | Al | | | Cd | | | Co | | | Cr | | | Cu | | | Fe | | |
| --- | --- | --- | --- | --- | --- | --- | --- | --- | --- | --- | --- | --- | --- | --- | --- | --- | --- | --- |
|  | Max | Min | Av | Max | Min | Av | Max | Min | Av | Max | Min | Av | Max | Min | Av | Max | Min | Av |
| Urban (Riyadh) | | | | | | | | | | | | | | | |  |  |  |
| Bulk | 12700 | 2450 | 5405 | 0.10 | 0.00 | 0.012 | 0.00 | 0.00 | 0.00 | 31.4 | 1.49 | 9.08 | 4.41 | 0.00 | 0.702 | 9990 | 2760 | 4710 |
| 250-63 | 12300 | 3630 | 6260 | 0.40 | 0.00 | 0.070 | 4.41 | 0.00 | 1.29 | 31.3 | 7.56 | 15.2 | 12.6 | 2.18 | 5.88 | 9330 | 3650 | 5760 |
| ≤63 | 11800 | 3580 | 7860 | 0.20 | 0.00 | 0.012 | 5.94 | 0.93 | 3.45 | 36.6 | 10.9 | 24.0 | 23.8 | 4.22 | 11.2 | 11700 | 5060 | 8780 |
|  | Mn | | | Ni | | | Pb | | | Ti | | | Zn | | |  |  |  |
| Bulk | 166 | 49.7 | 81.5 | 23.9 | 0.00 | 4.41 | 12.6 | 1.28 | 5.68 | 240 | 24.5 | 117 | 31.3 | 4.69 | 13.7 |  |  |  |
| 250-63 | 156 | 53.0 | 92.5 | 29.2 | 4.73 | 12.4 | 14.7 | 2.65 | 6.29 | 241 | 53.3 | 138 | 46.4 | 11.7 | 22.1 |  |  |  |
| ≤63 | 194 | 73.8 | 139 | 32.4 | 7.82 | 19.7 | 11.8 | 4.24 | 7.78 | 331 | 46.7 | 188 | 62.4 | 23.4 | 33.5 |  |  |  |
| Suburban | | | | | | | | | | | | | | | | | | |
|  | Al | | | Cd | | | Co | | | Cr | | | Cu | | | Fe | | |
| Bulk | 7650 | 3060 | 5360 | 0.00 | 0.00 | 0.00 | 2.22 | 0.00 | 1.11 | 5.94 | 4.45 | 5.19 | 2.59 | 0.00 | 1.30 | 9650 | 3600 | 6630 |
| 250-63 | 8530 | 4500 | 6520 | 0.00 | 0.00 | 0.00 | 1.59 | 1.59 | 1.59 | 14.6 | 13.9 | 14.2 | 3.62 | 2.94 | 3.28 | 8880 | 5350 | 7120 |
| ≤63 | 17000 | 9250 | 13100 | 0.10 | 0.00 | 0.05 | 6.04 | 4.89 | 5.47 | 40.7 | 29.6 | 35.1 | 11.1 | 10.5 | 10.8 | 16700 | 10200 | 13500 |
|  | Mn | | | Ni | | | Pb | | | Ti | | | Zn | | |  |  |  |
| Bulk | 163 | 58.6 | 111 | 9.63 | 3.04 | 6.34 | 4.96 | 4.38 | 4.67 | 200 | 113 | 156 | 19.24 | 3.36 | 11.3 |  |  |  |
| 250-63 | 148 | 74.2 | 111 | 13.6 | 9.83 | 11.71 | 5.47 | 5.16 | 5.32 | 198 | 149 | 174 | 17.8 | 11.2 | 14.5 |  |  |  |
| ≤63 | 291 | 167 | 229 | 27.2 | 26.1 | 26.64 | 10.6 | 7.60 | 9.10 | 552 | 371 | 4614 | 35.9 | 23.3 | 29.6 |  |  |  |

**Table S3b** Minimum, maximum and average concentrations of the heavy metals (mg kg^-1^) in the different particle size fractions of urban and suburban soils at mining area of Mahad AD’Dahab.

| Soil fraction µm | Al | | | Cd | | | Co | | | Cr | | | Cu | | | Fe | | |
| --- | --- | --- | --- | --- | --- | --- | --- | --- | --- | --- | --- | --- | --- | --- | --- | --- | --- | --- |
|  | Max | Min | Av | Max | Min | Av | Max | Min | Av | Max | Min | Av | Max | Min | Av | Max | Min | Av |
| Urban (Riyadh) | | | | | | | | | | | | | | | |  |  |  |
| 2000 | 17800 | 10100 | 13950 | 0.395 | 0.000 | 0.197 | 0.546 | 0.000 | 0.273 | 18.4 | 7.98 | 13.2 | 32.8 | 4.12 | 18.5 | 15100 | 10600 | 12850 |
| 250-63 | 15200 | 9760 | 12480 | 0.397 | 0.000 | 0.199 | 8.16 | 5.55 | 6.85 | 27.6 | 19.5 | 23.6 | 43.8 | 13.9 | 28.9 | 16600 | 12900 | 14750 |
| 63 | 23700 | 16700 | 20200 | 1.29 | 0.099 | 0.693 | 12.0 | 10.4 | 11.2 | 41.9 | 32.7 | 37.3 | 104 | 56.3 | 80.4 | 20900 | 20500 | 20700 |
|  | Mn | | | Ni | | | Pb | | | Ti | | | Zn | | |  |  |  |
| 2000 | 392 | 223 | 308 | 14.3 | 2.38 | 8.37 | 22.2 | 5.57 | 13.9 | 613 | 563 | 588 | 90.0 | 18.5 | 54.3 |  |  |  |
| 250-63 | 354 | 250 | 302 | 26.0 | 14.7 | 20.3 | 25.8 | 5.92 | 15.9 | 627 | 531 | 579 | 96.4 | 26.9 | 61.6 |  |  |  |
| 63 | 450 | 416 | 433 | 40.3 | 25.9 | 33.1 | 56.2 | 17.9 | 37.1 | 1222 | 464 | 843 | 297 | 106 | 202 |  |  |  |
| Suburban | | | | | | | | | | | | | | | | | | |
|  | Al | | | Cd | | | Co | | | Cr | | | Cu | | | Fe | | |
| 2000 | 12800 | 8480 | 10640 | 0.000 | 0.000 | 0.000 | 0.000 | 0.000 | 0.000 | 12.6 | 5.94 | 9.27 | 17.3 | 6.97 | 12.1 | 14600 | 10300 | 12450 |
| 250-63 | 9740 | 8530 | 9135 | 0.000 | 0.000 | 0.000 | 7.53 | 6.30 | 6.91 | 21.5 | 15.0 | 18.3 | 19.0 | 14.6 | 16.8 | 15200 | 12600 | 13900 |
| 63 | 16000 | 14600 | 15300 | 0.000 | 0.000 | 0.000 | 10.9 | 10.2 | 10.5 | 35.7 | 34.4 | 35.0 | 35.9 | 32.2 | 34.1 | 20100 | 19800 | 19950 |
|  | Mn | | | Ni | | | Pb | | | Ti | | | Zn | | |  |  |  |
| 2000 | 378 | 234 | 306 | 10.9 | 3.80 | 7.35 | 6.28 | 4.39 | 5.34 | 987 | 631 | 809 | 38.5 | 17.1 | 27.8 |  |  |  |
| 250-63 | 302 | 268 | 285 | 22.2 | 18.4 | 20.3 | 8.46 | 7.31 | 7.89 | 788 | 702 | 745 | 36.7 | 30.7 | 33.7 |  |  |  |
| 63 | 368 | 357 | 363 | 34.9 | 34.4 | 34.7 | 12.9 | 12.4 | 12.7 | 891 | 720 | 806 | 70.6 | 63.7 | 67.2 |  |  |  |

**Table S4** Pearson correlation between heavy metals concentration in dust and soil less than 63 µm overall investigated areas.

|  |  |  |  |  |  | Dust |  |  |  |  |  |  |
| --- | --- | --- | --- | --- | --- | --- | --- | --- | --- | --- | --- | --- |
|  | Metal | Al | Cd | Co | Cr | Cu | Fe | Mn | Ni | Pb | Ti | Zn |
| Soil | Al | 0.41 | **0.48** | 0.30 | -0.22 | **0.57** | **0.58** | **0.69** | -0.08 | 0.35 | **0.74** | **0.45** |
|  | Cd | 0.41 | **0.55*** | 0.36 | -0.12 | **0.58** | **0.63** | **0.72** | 0.05 | **0.49** | **0.72** | 0.40 |
|  | Co | 0.36 | **0.43** | 0.30 | -0.26 | **0.57** | **0.51** | **0.64** | -0.09 | 0.27 | **0.72** | **0.43** |
|  | Cr | 0.36 | 0.35 | 0.16 | -0.11 | 0.36 | 0.42 | **0.46** | -0.01 | 0.16 | **0.47** | 0.38 |
|  | Cu | **0.42** | **0.58** | 0.41 | -0.22 | **0.67*** | **0.66** | **0.79** | -0.02 | **0.49** | **0.84** | **0.44** |
|  | Fe | 0.36 | **0.46** | 0.30 | -0.27 | **0.57** | **0.53*** | **0.65** | -0.13 | 0.31 | **0.74** | 0.42 |
|  | Mn | 0.33 | **0.47** | 0.32 | -0.30 | **0.58** | **0.53** | **0.66*** | -0.15 | 0.31 | **0.76** | 0.40 |
|  | NI | 0.40 | **0.44** | 0.21 | -0.08 | **0.43** | **0.47** | **0.52** | 0.06 | 0.27 | **0.53** | 0.39 |
|  | Pb | **0.43** | **0.58** | 0.38 | -0.15 | **0.62** | **0.65** | **0.75** | 0.03 | **0.52*** | **0.78** | **0.44** |
|  | TI | 0.15 | 0.25 | 0.21 | -0.33 | 0.41 | 0.29 | 0.41 | -0.21 | 0.11 | **0.55*** | 0.24 |
|  | Zn | **0.43** | **0.57** | 0.39 | -0.17 | **0.64** | **0.66** | **0.77** | 0.01 | **0.51** | **0.80** | **0.44*** |

**Table S5** Average concentrations (mg kg^-1^) of heavy metals in dust of urban and suburban in relation with season.

| Site | Season | Heavy Metals | | | | | | | | | | |
| --- | --- | --- | --- | --- | --- | --- | --- | --- | --- | --- | --- | --- |
|  |  | Fe | Al | Ti | Cd | Co | Cr | Cu | Ni | Pb | Mn | Zn |
| Riyadh | | | | | | | | | | | | |
| Urban | Winter | 12800 | 10200 | 368 | 0.10 | 4.43 | 29.6 | 28.7 | 24.6 | 19.1 | 205 | 525 |
|  | Spring | 15000 | 12700 | 616 | 0.12 | 5.74 | 34.1 | 21.2 | 32.0 | 13.2 | 252 | 616 |
|  | Summer | 12200 | 9640 | 321 | 0.05 | 4.08 | 27.5 | 18.2 | 25.5 | 10.2 | 198 | 178 |
|  | Autumn | 11700 | 9270 | 297 | 0.12 | 3.75 | 27.6 | 27.8 | 24.6 | 22.1 | 187 | 1080 |
| Suburban | Winter | 13500 | 11400 | 398 | 0.75 | 1.34 | 28.0 | 7.97 | 23.8 | 13.4 | 205 | 670 |
|  | Spring | 14400 | 12300 | 418 | 0.00 | 3.20 | 31.6 | 13.8 | 27.9 | 8.45 | 234 | 817 |
|  | Summer | 8690 | 6650 | 211 | 0.00 | 2.66 | 17.8 | 7.76 | 16.6 | 6.59 | 137 | 115 |
|  | Autumn | 9600 | 7470 | 226 | 0.00 | 3.03 | 20.0 | 10.4 | 19.3 | 6.01 | 139 | 809 |
| Mahad AD’Dahab | | | | | | | | | | | | |
| Urban | Winter | 20100 | 15000 | 947 | 0.694 | 8.32 | 31.4 | 135 | 29.8 | 43.9 | 375 | 1020 |
|  | Spring | 18300 | 13800 | 755 | 0.430 | 8.79 | 33.2 | 131 | 33.1 | 34.5 | 355 | 1240 |
|  | Summer | 17100 | 12700 | 720 | 0.863 | 8.04 | 26.2 | 133 | 26.1 | 41.8 | 341 | 600 |
|  | Autumn | 18500 | 14200 | 791 | 0.775 | 8.43 | 29.3 | 108 | 28.3 | 34.9 | 359 | 1070 |
| Suburban | Winter | 14500 | 9850 | 586 | 0.132 | 4.69 | 17.9 | 34.8 | 19.9 | 9.66 | 279 | 763 |
|  | Spring | 17700 | 12900 | 682 | 0.132 | 5.92 | 22.1 | 26.5 | 23.7 | 7.44 | 368 | 779 |
|  | Summer | 13600 | 9020 | 561 | 0.132 | 4.05 | 16.9 | 17.0 | 16.9 | 7.22 | 257 | 323 |
|  | Autumn | 14000 | 9030 | 538 | 0.099 | 4.18 | 17.5 | 18.9 | 19.3 | 5.37 | 271 | 800 |

**Table S6** The Pearson correlation coefficients (r) among various metals at dust sampling in Riyadh and Mahad AD’Dahab area.

| Riyadh area | | | | | | | | | | | |
| --- | --- | --- | --- | --- | --- | --- | --- | --- | --- | --- | --- |
| Metal | Al | Cd | Co | Cr | Cu | Fe | Mn | Ni | Pb | Ti | Zn |
| Al | 1.00 |  |  |  |  |  |  |  |  |  |  |
| Cd | 0.10 | 1.00 |  |  |  |  |  |  |  |  |  |
| Co | 0.59* | 0.69* | 1.00 |  |  |  |  |  |  |  |  |
| Cr | 0.93* | 0.23 | 0.75* | 1.00 |  |  |  |  |  |  |  |
| Cu | 0.26 | -0.17 | 0.19 | 0.34 | 1.00 |  |  |  |  |  |  |
| Fe | 0.91* | 0.05 | 0.66* | 0.96* | 0.40 | 1.00 |  |  |  |  |  |
| Mn | 0.89* | -0.03 | 0.63* | 0.94* | 0.45 | 0.97* | 1.00 |  |  |  |  |
| Ni | 0.85* | 0.32 | 0.79* | 0.96* | 0.33 | 0.90* | 0.88* | 1.00 |  |  |  |
| Pb | 0.56* | 0.30 | 0.59* | 0.69* | 0.45 | 0.64* | 0.59* | 0.64* | 1.00 |  |  |
| Ti | 0.79* | 0.03 | 0.58* | 0.78* | 0.28 | 0.81* | 0.76* | 0.66* | 0.62* | 1.00 |  |
| Zn | 0.78* | -0.13 | 0.31 | 0.76* | 0.24 | 0.78* | 0.75* | 0.68* | 0.43 | 0.47* | 1.00 |
| Mahad AD’Dahab | | | | | | | | | | | |
| Metal | Al | Cd | Co | Cr | Cu | Fe | Mn | Ni | Pb | Ti | Zn |
| Al | 1.00 |  |  |  |  |  |  |  |  |  |  |
| Cd | 0.35 | 1.00 |  |  |  |  |  |  |  |  |  |
| Co | 0.96* | 0.35 | 1.00 |  |  |  |  |  |  |  |  |
| Cr | 0.97* | 0.46 | 0.97* | 1.00 |  |  |  |  |  |  |  |
| Cu | 0.60 | 0.93* | 0.65 | 0.73 | 1.00 |  |  |  |  |  |  |
| Fe | 0.97* | 0.37 | 0.86 | 0.92* | 0.57 | 1.00 |  |  |  |  |  |
| Mn | 0.96* | 0.19 | 0.84 | 0.87 | 0.41 | 0.98* | 1.00 |  |  |  |  |
| Ni | 0.99* | 0.42 | 0.97* | 0.99* | 0.68 | 0.95* | 0.92* | 1.00 |  |  |  |
| Pb | 0.50 | 0.98* | 0.53 | 0.61 | 0.97* | 0.49 | 0.32 | 0.57 | 1.00 |  |  |
| Ti | 0.94* | 0.61 | 0.88* | 0.96* | 0.81 | 0.94* | 0.86 | 0.96* | 0.72 | 1.00 |  |
| Zn | 0.78 | 0.76 | 0.69 | 0.82 | 0.85 | 0.84 | 0.72 | 0.81 | 0.80 | 0.95* | 1.00 |

**Table S7** PCA for heavy metals in dust samples of Riyadh and Mahad AD’Dahab.

|  | Riyadh area | | | | Mahad AD’Dahab area | |
| --- | --- | --- | --- | --- | --- | --- |
|  | F1 | F2 | F3 | F4 | F1 | F2 |
| Al | 0.95* | -0.02 | -0.01 | 0.14 | 0.95* | 0.19 |
| Cd | 0.00 | -0.09 | 0.95* | 0.00 | 0.08 | 0.95* |
| Co | 0.73* | 0.19 | 0.43 | -0.15 | 0.91* | 0.24 |
| Cr | 0.94* | 0.14 | 0.06 | 0.25 | 0.92* | 0.32 |
| Cu | 0.10 | 0.94* | -0.16 | 0.14 | 0.36 | 0.90* |
| Fe | 0.96* | 0.09 | -0.01 | 0.16 | 0.95* | 0.22 |
| Mn | 0.96* | 0.06 | -0.06 | 0.08 | 0.96* | 0.04 |
| Ni | 0.92* | 0.06 | 0.15 | 0.17 | 0.95* | 0.23 |
| Pb | 0.11 | 0.64 | 0.33 | 0.59 | 0.26 | 0.95* |
| Ti | 0.86* | 0.11 | -0.04 | 0.05 | 0.79* | 0.46 |
| Zn | 0.28 | 0.19 | -0.08 | 0.91* | 0.69 | 0.40 |
| Eigenval | 6.28 | 1.73 | 1.23 | 0.66 | 7.88 | 1.98 |
| %total of variance | 57.12 | 15.75 | 11.21 | 5.98 | 71.61 | 18.03 |
| Cumul. % | 57.12 | 72.87 | 84.08 | 90.06 | 71.61 | 89.63 |

**Table S8** Average of hazard Quotient (HQ) for pathways exposure and for all categories of human of dust in urban and suburban areas in Riyadh.

| Hazard Quotient (HQ) | | | | | | | | | |
| --- | --- | --- | --- | --- | --- | --- | --- | --- | --- |
|  |  | Cd | Co | Cr | Cu | Mn | Ni | Pb | Zn |
|  | Children up to 6 years | |  |  |  |  |  |  |  |
| HQ_ing_ | Urban | 9.59E-04 | 1.79E-01 | 1.18E-01 | 7.16E-03 | 1.79E-02 | 1.59E-02 | 5.51E-02 | 2.39E-02 |
|  | Suburban | 0.00E+00 | 1.02E-01 | 9.70E-02 | 2.98E-03 | 1.53E-02 | 1.31E-02 | 2.95E-02 | 2.40E-02 |
| HQ_der_ | Urban | 2.68E-04 | 1.25E-03 | 6.36E-02 | 5.00E-05 | 1.25E-04 | 2.78E-03 | 3.85E-04 | 1.67E-04 |
|  | Suburban | 0.00E+00 | 7.11E-04 | 5.21E-02 | 2.08E-05 | 1.06E-04 | 2.28E-03 | 2.06E-04 | 1.68E-04 |
|  | Children up to 6-12 years | |  |  |  |  |  |  |  |
| HQ_ing_ | Urban | 2.64E-04 | 4.95E-02 | 3.27E-02 | 1.97E-03 | 4.95E-03 | 4.39E-03 | 1.52E-02 | 6.58E-03 |
|  | Suburban | 0.00E+00 | 2.81E-02 | 2.67E-02 | 8.21E-04 | 4.21E-03 | 3.60E-03 | 8.12E-03 | 6.63E-03 |
| HQ_der_ | Urban | 2.22E-04 | 1.04E-03 | 5.27E-02 | 4.14E-05 | 1.04E-04 | 2.30E-03 | 3.18E-04 | 1.38E-04 |
|  | Suburban | 0.00E+00 | 5.89E-04 | 4.31E-02 | 1.72E-05 | 8.81E-05 | 1.89E-03 | 1.70E-04 | 1.39E-04 |
|  | Adult |  |  |  |  |  |  |  |  |
| HQ_ing_ | Urban | 5.48E-05 | 1.02E-02 | 6.77E-03 | 4.09E-04 | 1.03E-03 | 9.10E-04 | 3.15E-03 | 1.36E-03 |
|  | Suburban | 0.00E+00 | 5.82E-03 | 5.54E-03 | 1.70E-04 | 8.72E-04 | 7.46E-04 | 1.68E-03 | 1.37E-03 |
| HQ_der_ | Urban | 5.57E-05 | 2.60E-04 | 1.32E-02 | 1.04E-05 | 2.61E-05 | 5.78E-04 | 8.00E-05 | 3.46E-05 |
|  | Suburban | 0.00E+00 | 1.48E-04 | 1.08E-02 | 4.32E-06 | 2.22E-05 | 4.74E-04 | 4.28E-05 | 3.49E-05 |
| HQ_ing_: Hazard quotient by ingestion; HQ_der_: Hazard quotient by intake dermal | | | | | | | | | |

**Table S9** Average of hazard Quotient (HQ) for pathways exposure and for all categories of human of dust in urban and suburban areas in Mahad AD’Dahab.

| Hazard Quotient (HQ) | | | | | | | | | |
| --- | --- | --- | --- | --- | --- | --- | --- | --- | --- |
|  |  | Cd | Co | Cr | Cu | Mn | Ni | Pb | Zn |
|  | Children up to 6 years | |  |  |  |  |  |  |  |
| HQ_ing_ | Urban | 8.25E-03 | 3.34E-01 | 1.20E-01 | 3.79E-02 | 3.05E-02 | 1.75E-02 | 1.32E-01 | 3.91E-02 |
|  | Suburban | 1.48E-03 | 1.88E-01 | 7.41E-02 | 7.25E-03 | 2.51E-02 | 1.19E-02 | 2.54E-02 | 2.65E-02 |
| HQ_der_ | Urban | 2.30E-03 | 2.33E-03 | 6.43E-02 | 2.65E-04 | 2.13E-04 | 3.06E-03 | 9.24E-04 | 2.73E-04 |
|  | Suburban | 4.14E-04 | 1.31E-03 | 3.98E-02 | 5.06E-05 | 1.75E-04 | 2.08E-03 | 1.77E-04 | 1.85E-04 |
|  | Children up to 6-12 years | |  |  |  |  |  |  |  |
| HQ_ing_ | Urban | 2.28E-03 | 9.23E-02 | 3.30E-02 | 1.05E-02 | 8.42E-03 | 4.84E-03 | 3.65E-02 | 1.08E-02 |
|  | Suburban | 4.09E-04 | 5.18E-02 | 2.04E-02 | 2.00E-03 | 6.92E-03 | 3.29E-03 | 6.99E-03 | 7.32E-03 |
| HQ_der_ | Urban | 1.91E-03 | 1.93E-03 | 5.32E-02 | 2.19E-04 | 1.76E-04 | 2.53E-03 | 7.65E-04 | 2.26E-04 |
|  | Suburban | 3.42E-04 | 1.08E-03 | 3.29E-02 | 4.19E-05 | 1.45E-04 | 1.72E-03 | 1.46E-04 | 1.53E-04 |
|  | Adult |  |  |  |  |  |  |  |  |
| HQ_ing_ | Urban | 4.72E-04 | 1.91E-02 | 6.84E-03 | 2.17E-03 | 1.74E-03 | 1.00E-03 | 7.57E-03 | 2.23E-03 |
|  | Suburban | 8.47E-05 | 1.07E-02 | 4.23E-03 | 4.15E-04 | 1.43E-03 | 6.82E-04 | 1.45E-03 | 1.52E-03 |
| HQ_der_ | Urban | 4.79E-04 | 4.86E-04 | 1.34E-02 | 5.50E-05 | 4.43E-05 | 6.37E-04 | 1.92E-04 | 5.68E-05 |
|  | Suburban | 8.61E-05 | 2.72E-04 | 8.27E-03 | 1.05E-05 | 3.64E-05 | 4.33E-04 | 3.68E-05 | 3.85E-05 |
| HQ_ing_: Hazard quotient by ingestion; HQ_der_: Hazard quotient by intake dermal | | | | | | | | | |

**Table S10** Average hazard index (HI) of dust in urban and suburban areas in Riyadh and Mahad AD’Dahab.

| Hazard Index (HI) | | | | | | | | | | |
| --- | --- | --- | --- | --- | --- | --- | --- | --- | --- | --- |
|  |  | Cd | Co | Cr | Cu | Mn | Ni | Pb | Zn | Sum |
|  | Children up to 6 years | |  |  |  |  |  |  |  |  |
| Riyadh | Urban | 1.23E-03 | 1.81E-01 | 1.82E-01 | 7.21E-03 | 1.81E-02 | 1.87E-02 | 5.55E-02 | 2.40E-02 | 4.87E-01 |
|  | Suburban | 0.00E+00 | 1.03E-01 | 1.49E-01 | 3.00E-03 | 1.54E-02 | 1.53E-02 | 2.97E-02 | 2.42E-02 | 3.39E-01 |
| Mahad AD'Dahab | Urban | 1.06E-02 | 3.37E-01 | 1.84E-01 | 3.82E-02 | 3.07E-02 | 2.06E-02 | 1.33E-01 | 3.94E-02 | 7.94E-01 |
|  | Suburban | 1.90E-03 | 1.89E-01 | 1.14E-01 | 7.30E-03 | 2.53E-02 | 1.40E-02 | 2.55E-02 | 2.67E-02 | 4.04E-01 |
|  | Children up to 6-12 years | |  |  |  |  |  |  |  |  |
| Riyadh | Urban | 4.86E-04 | 5.05E-02 | 8.53E-02 | 2.02E-03 | 5.05E-03 | 6.70E-03 | 1.55E-02 | 6.72E-03 | 1.72E-01 |
|  | Suburban | 0.00E+00 | 2.87E-02 | 6.98E-02 | 8.39E-04 | 4.30E-03 | 5.49E-03 | 8.29E-03 | 6.77E-03 | 1.24E-01 |
| Mahad AD'Dahab | Urban | 4.18E-03 | 9.42E-02 | 8.62E-02 | 1.07E-02 | 8.60E-03 | 7.37E-03 | 3.73E-02 | 1.10E-02 | 2.60E-01 |
|  | Suburban | 7.51E-04 | 5.29E-02 | 5.33E-02 | 2.04E-03 | 7.07E-03 | 5.02E-03 | 7.14E-03 | 7.47E-03 | 1.36E-01 |
|  | Adult |  |  |  |  |  |  |  |  |  |
| Riyadh | Urban | 1.10E-04 | 1.05E-02 | 2.00E-02 | 4.19E-04 | 1.05E-03 | 1.49E-03 | 3.23E-03 | 1.40E-03 | 3.82E-02 |
|  | Suburban | 0.00E+00 | 5.97E-03 | 1.64E-02 | 1.74E-04 | 8.94E-04 | 1.22E-03 | 1.73E-03 | 1.41E-03 | 2.78E-02 |
| Mahad AD'Dahab | Urban | 9.51E-04 | 1.96E-02 | 2.02E-02 | 2.22E-03 | 1.79E-03 | 1.64E-03 | 7.76E-03 | 2.29E-03 | 5.65E-02 |
|  | Suburban | 1.71E-04 | 1.10E-02 | 1.25E-02 | 4.25E-04 | 1.47E-03 | 1.12E-03 | 1.49E-03 | 1.56E-03 | 2.97E-02 |

**Table S11** Average of hazard Quotient (HQ) for pathways exposure and for all categories of human of soil in urban and suburban areas in Riyadh.

|  |  | Hazard Quotient (HQ) | | | | | | | |
| --- | --- | --- | --- | --- | --- | --- | --- | --- | --- |
|  |  | Cd | Co | Cr | Cu | Mn | Ni | Pb | Zn |
|  |  | Children up to 6 years | |  |  |  |  |  |  |
| HQ_ing_ | Urban | 1.48E-04 | 1.38E-01 | 9.55E-02 | 3.35E-03 | 1.18E-02 | 1.18E-02 | 2.66E-02 | 1.34E-03 |
|  | Suburban | 5.92E-04 | 2.18E-01 | 1.40E-01 | 3.22E-03 | 1.96E-02 | 1.59E-02 | 3.11E-02 | 1.18E-03 |
| HQ_der_ | Urban | 4.13E-05 | 9.61E-04 | 5.13E-02 | 2.34E-05 | 8.26E-05 | 2.06E-03 | 1.85E-04 | 9.32E-06 |
|  | Suburban | 1.65E-04 | 1.52E-03 | 7.52E-02 | 2.25E-05 | 1.37E-04 | 2.78E-03 | 2.17E-04 | 8.23E-06 |
|  |  | Children up to 6-12 years | |  |  |  |  |  |  |
| HQ_ing_ | Urban | 4.08E-05 | 3.80E-02 | 2.64E-02 | 9.25E-04 | 3.27E-03 | 3.25E-03 | 7.33E-03 | 3.68E-04 |
|  | Suburban | 1.63E-04 | 6.01E-02 | 3.86E-02 | 8.89E-04 | 5.40E-03 | 4.39E-03 | 8.58E-03 | 3.25E-04 |
| HQ_der_ | Urban | 3.42E-05 | 7.95E-04 | 4.25E-02 | 1.94E-05 | 6.84E-05 | 1.70E-03 | 1.53E-04 | 7.71E-06 |
|  | Suburban | 1.37E-04 | 1.26E-03 | 6.22E-02 | 1.86E-05 | 1.13E-04 | 2.30E-03 | 1.80E-04 | 6.81E-06 |
|  |  | Adult |  |  |  |  |  |  |  |
| HQ_ing_ | Urban | 8.45E-06 | 7.87E-03 | 5.46E-03 | 1.92E-04 | 6.76E-04 | 6.73E-04 | 1.52E-03 | 7.63E-05 |
|  | Suburban | 3.38E-05 | 1.24E-02 | 8.00E-03 | 1.84E-04 | 1.12E-03 | 9.10E-04 | 1.78E-03 | 6.74E-05 |
| HQ_der_ | Urban | 8.59E-06 | 2.00E-04 | 1.07E-02 | 4.87E-06 | 1.72E-05 | 4.28E-04 | 3.86E-05 | 1.94E-06 |
|  | Suburban | 3.44E-05 | 3.16E-04 | 1.56E-02 | 4.68E-06 | 2.84E-05 | 5.78E-04 | 4.51E-05 | 1.71E-06 |

**Table S12** Average of hazard Quotient (HQ) for pathways exposure and for all categories of human of soil in urban and suburban areas in Mahad AD’Dahab.

|  |  | Hazard Quotient (HQ) | | | | | | | |
| --- | --- | --- | --- | --- | --- | --- | --- | --- | --- |
|  |  | Cd | Co | Cr | Cu | Mn | Ni | Pb | Zn |
|  |  | Children up to 6 years | |  |  |  |  |  |  |
| HQ_ing_ | Urban | 8.29E-03 | 4.46E-01 | 1.49E-01 | 2.40E-02 | 3.70E-02 | 1.98E-02 | 1.27E-01 | 8.04E-03 |
|  | Suburban | 0.00E+00 | 4.20E-01 | 1.40E-01 | 1.02E-02 | 3.10E-02 | 2.07E-02 | 4.33E-02 | 2.68E-03 |
| HQ_der_ | Urban | 2.31E-03 | 3.12E-03 | 7.99E-02 | 1.68E-04 | 2.58E-04 | 3.45E-03 | 8.84E-04 | 5.61E-05 |
|  | Suburban | 0.00E+00 | 2.93E-03 | 7.50E-02 | 7.10E-05 | 2.16E-04 | 3.62E-03 | 3.02E-04 | 1.87E-05 |
|  |  | Children up to 6-12 years | |  |  |  |  |  |  |
| HQ_ing_ | Urban | 2.29E-03 | 1.23E-01 | 4.10E-02 | 6.63E-03 | 1.02E-02 | 5.45E-03 | 3.49E-02 | 2.22E-03 |
|  | Suburban | 0.00E+00 | 1.16E-01 | 3.85E-02 | 2.81E-03 | 8.54E-03 | 5.72E-03 | 1.19E-02 | 7.38E-04 |
| HQ_der_ | Urban | 1.91E-03 | 2.58E-03 | 6.61E-02 | 1.39E-04 | 2.14E-04 | 2.86E-03 | 7.32E-04 | 4.64E-05 |
|  | Suburban | 0.00E+00 | 2.42E-03 | 6.20E-02 | 5.88E-05 | 1.79E-04 | 2.99E-03 | 2.50E-04 | 1.55E-05 |
|  |  | Adult |  |  |  |  |  |  |  |
| HQ_ing_ | Urban | 4.73E-04 | 2.55E-02 | 8.50E-03 | 1.37E-03 | 2.11E-03 | 1.13E-03 | 7.24E-03 | 4.59E-04 |
|  | Suburban | 0.00E+00 | 2.40E-02 | 7.98E-03 | 5.82E-04 | 1.77E-03 | 1.18E-03 | 2.47E-03 | 1.53E-04 |
| HQ_der_ | Urban | 4.81E-04 | 6.48E-04 | 1.66E-02 | 3.49E-05 | 5.37E-05 | 7.18E-04 | 1.84E-04 | 1.17E-05 |
|  | Suburban | 0.00E+00 | 6.09E-04 | 1.56E-02 | 1.48E-05 | 4.49E-05 | 7.52E-04 | 6.28E-05 | 3.89E-06 |

**Table S13** Average hazard index (HI) of soil in urban and suburban areas in Riyadh and Mahad AD’Dahab.

|  |  | Hazard index (HI) | | | | | | | | |
| --- | --- | --- | --- | --- | --- | --- | --- | --- | --- | --- |
|  |  | Cd | Co | Cr | Cu | Mn | Ni | Pb | Zn | Sum |
|  |  | Children up to 6 years | |  |  |  |  |  |  |  |
| Riyadh | Urban | 1.89E-04 | 1.39E-01 | 1.47E-01 | 3.38E-03 | 1.19E-02 | 1.38E-02 | 2.67E-02 | 1.34E-03 | 3.43E-01 |
|  | Suburban | 7.58E-04 | 2.19E-01 | 2.15E-01 | 3.25E-03 | 1.97E-02 | 1.87E-02 | 3.13E-02 | 1.19E-03 | 5.09E-01 |
| Mahad AD'Dahab | Urban | 1.06E-02 | 4.49E-01 | 2.29E-01 | 2.42E-02 | 3.72E-02 | 2.32E-02 | 1.28E-01 | 8.09E-03 | 9.09E-01 |
|  | Suburban | 0.00E+00 | 4.23E-01 | 2.15E-01 | 1.02E-02 | 3.12E-02 | 2.43E-02 | 4.36E-02 | 2.70E-03 | 7.49E-01 |
|  |  | Children up to 6-12 years | |  |  |  |  |  |  |  |
| Riyadh | Urban | 7.50E-05 | 3.88E-02 | 6.88E-02 | 9.44E-04 | 3.33E-03 | 4.95E-03 | 7.48E-03 | 3.76E-04 | 1.25E-01 |
|  | Suburban | 3.00E-04 | 6.13E-02 | 1.01E-01 | 9.08E-04 | 5.51E-03 | 6.69E-03 | 8.76E-03 | 3.32E-04 | 1.85E-01 |
| Mahad AD'Dahab | Urban | 4.20E-03 | 1.26E-01 | 1.07E-01 | 6.77E-03 | 1.04E-02 | 8.31E-03 | 3.57E-02 | 2.26E-03 | 3.00E-01 |
|  | Suburban | 0.00E+00 | 1.18E-01 | 1.01E-01 | 2.87E-03 | 8.72E-03 | 8.71E-03 | 1.22E-02 | 7.54E-04 | 2.52E-01 |
|  |  | Adult |  |  |  |  |  |  |  |  |
| Riyadh | Urban | 1.70E-05 | 8.07E-03 | 1.61E-02 | 1.96E-04 | 6.94E-04 | 1.10E-03 | 1.56E-03 | 7.82E-05 | 2.78E-02 |
|  | Suburban | 6.83E-05 | 1.28E-02 | 2.36E-02 | 1.89E-04 | 1.15E-03 | 1.49E-03 | 1.82E-03 | 6.91E-05 | 4.12E-02 |
| Mahad AD'Dahab | Urban | 9.55E-04 | 2.62E-02 | 2.51E-02 | 1.41E-03 | 2.17E-03 | 1.85E-03 | 7.42E-03 | 4.71E-04 | 6.55E-02 |
|  | Suburban | 0.00E+00 | 2.46E-02 | 2.36E-02 | 5.96E-04 | 1.81E-03 | 1.94E-03 | 2.54E-03 | 1.57E-04 | 5.52E-02 |


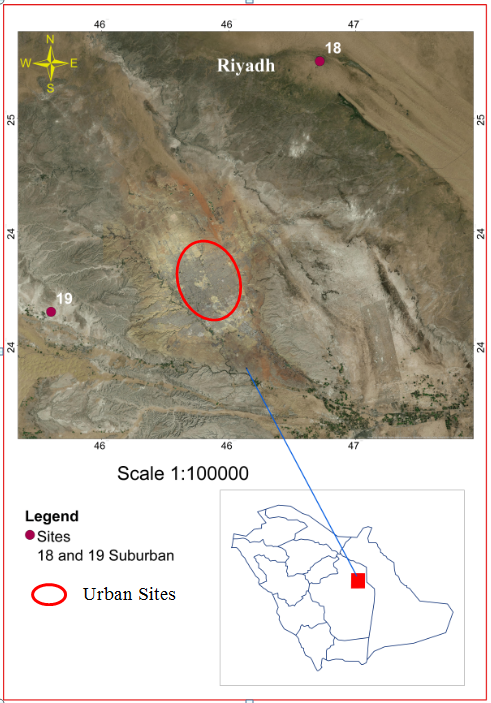


**Fig. S1a** Location map of the studied Riyadh areas. Landsat-8 images were used to construct this map. The map was created in ArcGIS 10.4.2 software developed by ESRI (Environmental Systems Research Institute). ArcGIS 10.4.2 software was downloaded from <https://arcview-gis.software.informer.com/10.4/>


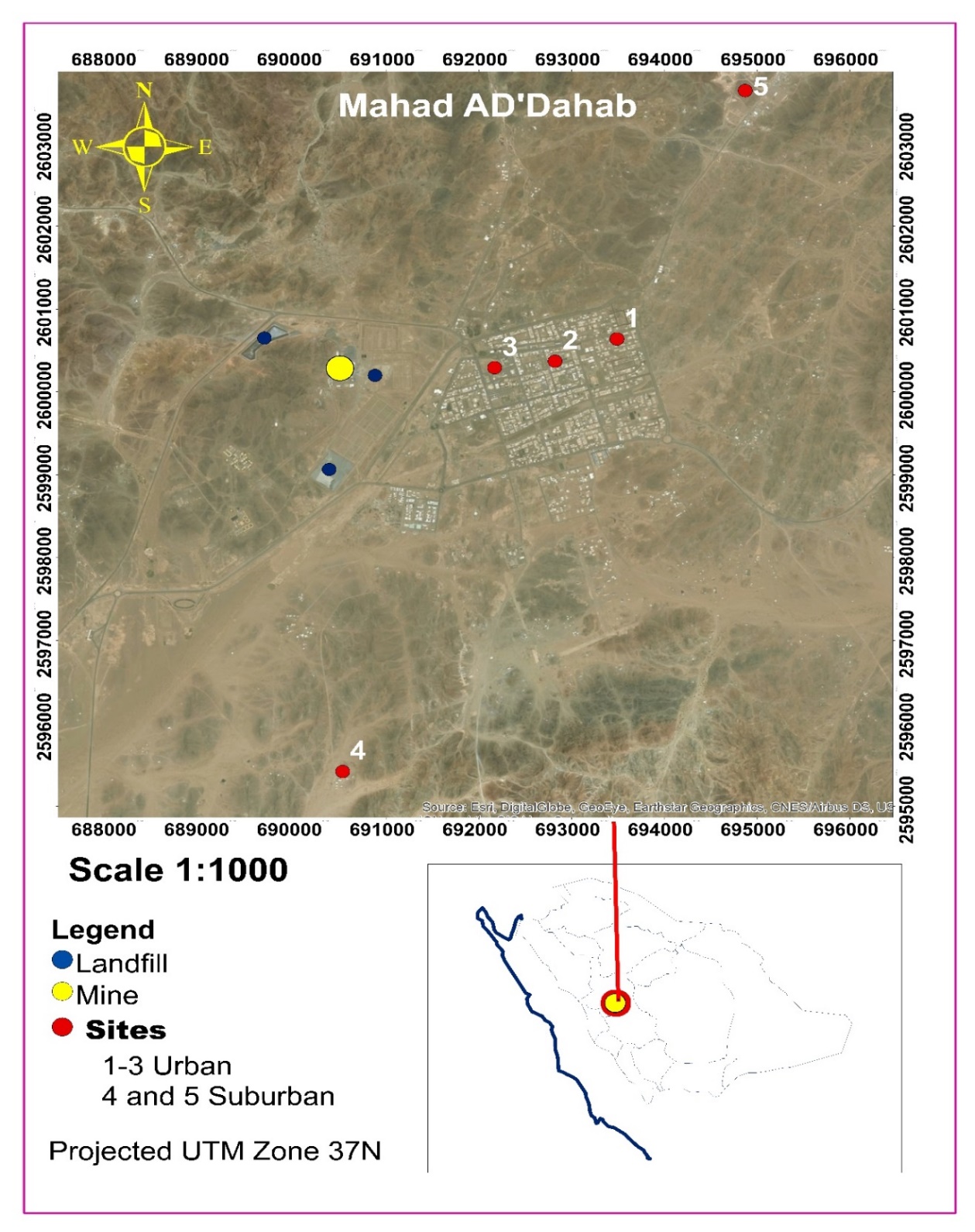


**Fig. S1b** Location map of the studied Mahad AD’Dahab areas. Landsat-8 images were used to construct this map. The map was created in ArcGIS 10.4.2 software developed by ESRI (Environmental Systems Research Institute). ArcGIS 10.4.2 software was downloaded from <https://arcview-gis.software.informer.com/10.4/>

**Fig. S2** A marble dust collector with dimensions of 52.5 x 31.5 cm.

**Fig. S3** Distribution factors (DF) for Al, Fe, Ti, and Mn in the particle size fraction of urban and suburban soils (R: Riyadh area; M: Mahad AD’Dahab).

**Fig. S4** Distribution factors (DF) for Cu, Cr, Pb, Zn and Ni in the particle size fraction of urban and suburban soils (R: Riyadh area; M: Mahad AD’Dahab).

**Fig. S5** The Hazard Index average in dust samples of Riyadh.

**Fig. S6** Average the Hazard Index in dust samples of Mahad AD’Dahab.

**Fig. S7** Total hazard index for ∑ human categories in dust samples of Riyadh (A), and Mahad AD’Dahab (B).

**Fig. S8** Average the hazard index (HI) in urban and suburban in dust samples of Riyadh and Mahad AD’Dahab.

**Fig. S9** The Hazard Index average in soil samples of Riyadh

**Fig. S10** The Hazard Index average in soil samples of Mahad AD’Dahab

**Fig. S11** Total hazard index for ∑ human categories in soil samples of Riyadh.

**Fig. S12** Total hazard index for ∑ human categories in soil samples of Mahad AD’Dahab.

**Fig. S13** Average the hazard index (HI) in urban and suburban in soil samples of Riyadh and Mahad AD’Dahab.
